# HDAC6 as a target for neurodegenerative diseases: what makes it different from the other HDACs?

**DOI:** 10.1186/1750-1326-8-7

**Published:** 2013-01-29

**Authors:** Claudia Simões-Pires, Vincent Zwick, Alessandra Nurisso, Esther Schenker, Pierre-Alain Carrupt, Muriel Cuendet

**Affiliations:** 1 School of Pharmaceutical Sciences, University of Geneva, University of Lausanne, Quai Ernest-Ansermet 30, CH-1211, Geneva 4, Switzerland; 2 Institut de Recherches Servier, Rue de la République 3, 92150, Suresnes, France

**Keywords:** Histone deacetylase, HDAC6, Neurodegenerative diseases

## Abstract

Histone deacetylase (HDAC) inhibitors have been demonstrated to be beneficial in animal models of neurodegenerative diseases. Such results were mainly associated with the epigenetic modulation caused by HDACs, especially those from class I, via chromatin deacetylation. However, other mechanisms may contribute to the neuroprotective effect of HDAC inhibitors, since each HDAC may present distinct specific functions within the neurodegenerative cascades. Such an example is HDAC6 for which the role in neurodegeneration has been partially elucidated so far. The strategy to be adopted in promising therapeutics targeting HDAC6 is still controversial. Specific inhibitors exert neuroprotection by increasing the acetylation levels of α-tubulin with subsequent improvement of the axonal transport, which is usually impaired in neurodegenerative disorders. On the other hand, an induction of HDAC6 would theoretically contribute to the degradation of protein aggregates which characterize various neurodegenerative disorders, including Alzheimer’s, Parkinson’s and Hutington’s diseases. This review describes the specific role of HDAC6 compared to the other HDACs in the context of neurodegeneration, by collecting *in silico*, *in vitro* and *in vivo* results regarding the inhibition and/or knockdown of HDAC6 and other HDACs. Moreover, structure, function, subcellular localization, as well as the level of HDAC6 expression within brain regions are reviewed and compared to the other HDAC isoforms. In various neurodegenerative diseases, the mechanisms underlying HDAC6 interaction with other proteins seem to be a promising approach in understanding the modulation of HDAC6 activity.

## Introduction

Histone deacetylases (HDACs) are enzymes that deacetylate lysine residues from histones as well as from several other nuclear, cytoplasmic and mitochondrial non-histone proteins. In mammals, 18 HDACs have been phylogenetically classified into four classes. Classes I, II, and IV belong to the Rpd3/Hda1 family [1]. Class I includes the constitutively expressed HDACs 1 to 3 and HDAC8 [2]. Class II is subdivided into classes IIa (HDAC4, 5, 7, and 9) and IIb (HDAC6 and 10). Enzymes from class IIa are able to shuttle between the cytosol and the nucleus, and show a weaker deacetylase activity [3]. Class IIb is mostly found in the cytosol with a preference for non-histone proteins [4], whereas HDAC11 is the sole member of class IV. These HDACs are usually referred as classical HDACs, whereas class III, called sirtuins, are NAD^+^ dependent enzymes with different structural features [5].

**Figure 1:**
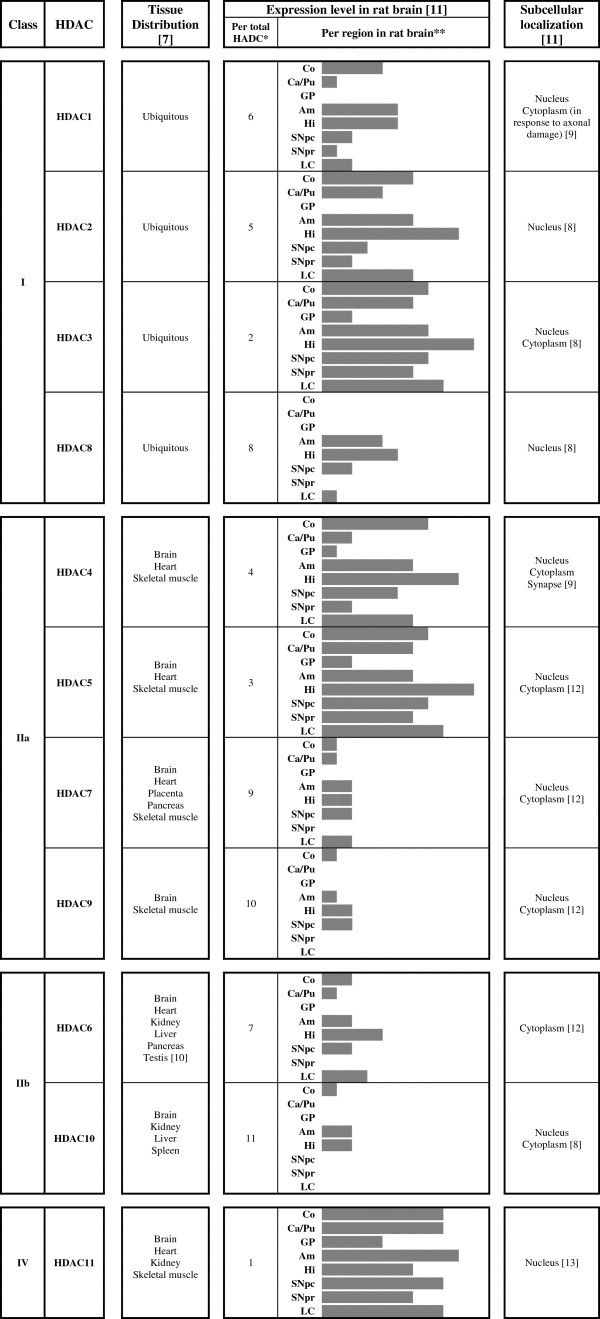
**HDAC isoforms distribution in tissues and rat brain regions, as well as their subcellular localization.** Am: Amigadala, As: Astrocytes, Ca/Pu: Caudate/Putamen, Co: Cortex, GP: Globus palidus, Hi: Hippocampus, LC: Locus coeruleus, Ne: neurons, Ol: oligodendrocytes, SNpc: Substantia nigra compacta, SNpr: Substantia nigra reticulata, VEC: Vessel endothelial cells; * classified from 1 (most expressed HDAC isoform) to 11 (less expressed HDAC isoform); ** diagrams are a graphical representation of the relative expression of each HDAC isoform in a scale from low to high (0–5), adapted from Broide et al. [11-13].

The role of HDACs has been studied within several cell processes based on phenotypic changes after isoform-specific knockdown or treatment with HDAC inhibitors. The consequences of an inhibition of HDACs may result in contradictory results, which seem to depend partially on cell type [6]. Knockout analyses of various class I and class II HDAC proteins suggested that class I HDACs are involved in cell proliferation and survival and are expressed ubiquitously in different body tissues, while class II HDACs seem to have tissue-specific roles [7,8]. Moreover, the specific role of each HDAC is directly related to their specific molecular substrates. To our knowledge, more than 50 non-histone proteins have been identified as substrates for HDACs [9]. On the basis of animal tissue expression and serial analysis of gene expression (SAGE) data from the human transcriptome map [7,10,11], the distribution of HDAC isoforms in body tissues is presented in Figure 1 together with their distribution in rat brain [11]. It is also important to notice that the level of expression may differ when specific pathologies are present, such as cancer, where some HDAC isoforms are overexpressed [10].

The deacetylase activity of HDACs is opposed to that of histone acetyl transferases (HATs) and several studies have demonstrated the relevance of the HDAC/HAT enzymatic balance in neuronal homeostasis [14]. This balance is involved in neurophysiological functions, memory processes and learning. A deregulation of HDAC/HAT activity has been observed in several neurodegenerative diseases (NDs), and a decrease in histone acetylation levels may affect the expression of genes involved in apoptosis and neuroprotection [14-16].

Several reviews discussed the importance of various HDACs in specific NDs [17,18] and recently, HDAC6 was suggested to be a promising target for some of them [19]. In the present work, we aim at reviewing the published data regarding HDAC6. The structural and functional features of this specific isoform are compared to other classical HDACs. The specific role of HDAC6 in NDs is discussed and the impact of HDAC6 modulation via inhibition, induction and interaction with other proteins in various diseases such as Alzheimer’s (AD), Parkinson’s (PD) and Huntington’s (HD), frontotemporal dementia (FTLD), amyotrophic lateral sclerosis (ALS) and Charcot-Marie-Tooth disease (CMT) is discussed.

## Structural differences between HDACs: what makes HDAC6 different from the others

The bacterial HDAC homologue HDLP from *Aquifex aeolicus* was the first HDAC-like protein structure solved by X-ray in 1999 [20]. Alignment studies combined with structural analyses revealed the presence of a conserved 11 Å deep channel among all HDAC structures, with a zinc ion located at the bottom [21-23]. The zinc-dependent catalytic action consists in the removal of acetyl groups from lysine residues belonging to histone or non-histone proteins [24]. Even if a certain degree of homology in the catalytic domains was found, the so-called zinc-dependent HDACs have been classified into three families (classes I, II and IV) depending on their primary sequence similarity to homologous enzymes from *Saccharomyces cerevisiae*[25].

Class I is characterized by four ubiquitous and relatively small enzymes (∼500 amino acids) essentially located in the nucleus of cells [25,26]. HDACs 1 to 3 are found in complexes with specific transcriptional co-repressors, blocking the expression of tumor suppressor genes [27]. Interestingly, these enzymes share an internal dynamic cavity adjacent to the catalytic pocket that seems to facilitate the egress of the enzymatic products from the active site [20,22]. Another nuclear zinc-dependent HDAC, HDAC11, was found to be closely related to class I. However, this enzyme did not show enough identity to this class to be placed in it and a new class (IV) was proposed with HDAC11 as the only member [10].

Class II consists of six larger enzymes (∼1000 amino acids) that can be further classified according to their sequence homology and domain organization into classes IIa and IIb [10]. The N-terminal domain found in class IIa members is the one responsible for nuclear-cytoplasmic shuttling through a phosphorylation-dependent binding to specific 14-3-3 proteins. Such interactions regulate the activity of transcription factors such as the myocyte enhancer factor-2 (MEF2), which exerts a repressor role in a variety of biological functions, from myogenesis to Epstein-Barr virus transcriptional regulation [27]. Moreover, this class shows another zinc ion coordinated to a Cys-Cys-His-Cys motif close to the cavity that may participate in substrate recognition or in protein interactions [28].

The presence of two catalytic domains in HDAC6 allows this isoform to be classified into class IIb together with HDAC10. While HDAC10 has catalytically inactive domains whose biological function is still unknown, HDAC6 was shown to take part in the microtubule network by acting as a specific α-tubulin deacetylase. Moreover, HDAC6 was able to deacetylate other substrates and to bind ubiquitin, thus modulating cell protective response to cytotoxic accumulation of misfolded and aggregated proteins [19,29,30].

**Figure 2:**
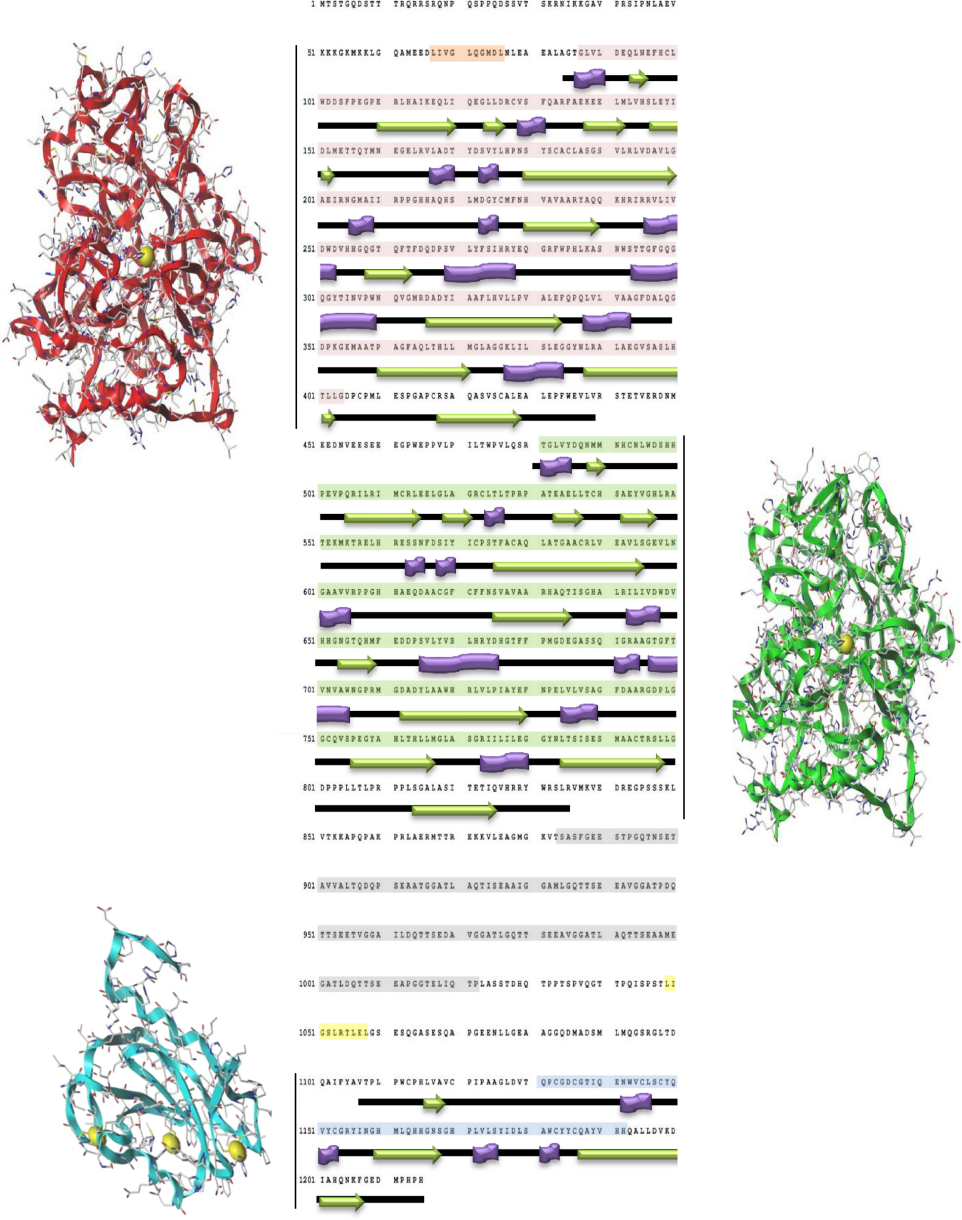
**HDAC6 domain organization.** Catalytic Domain I (CDI) primary sequence is highlighted in pale red; CDI three-dimensional structure obtained by homology modeling techniques by using HDAC7 x-ray structure as a template is represented with red ribbons. Catalytic Domain II (CDII) primary sequence is highlighted in pale green; CDII three-dimensional structure obtained by homology modeling techniques by using HDAC7 x-ray structure as a template is represented with green ribbons. Primary sequence of HDAC6 ubiquitin binding domain (ZnFUBP) is highlighted in blue whereas its three-dimensional structure (PDB ID 3C5K) is represented with cyan ribbons. Sequences corresponding to the tetradecapeptide repeating domain (SE14) and to the nuclear export domains NES1, NES2 are highlighted in pale gray, orange and yellow, respectively. Information about HDAC6 CD I/II and ZnFUBP secondary structures was retrieved from the human HDAC7 (PDB ID 3C10) and HDAC6 (PDB ID 3C5K) x-ray structures.

To be fully understood, the biological role of HDAC6 requires a deep structural knowledge. The 1215 amino acid residues characterizing the human HDAC6 are arranged in the space to form two independent catalytic domains with a zinc finger ubiquitin-binding domain located at the C-terminus [20]. In the enzymatic structure, it is also important to highlight the presence of a zone characterized by a Ser-Glu containing a tetradecapeptide repeating domain (SE14) responsible for HDAC6 intracellular retention and tau interaction. There are as well two leucine-rich nuclear export sequences (NES1, NES2), which play an essential role in the cytoplasmic/nuclear shuttling process [31,32] (Figure 2). What makes HDAC6 unique among all HDAC enzymes is the presence of the C-terminal zinc finger domain able to recognize unanchored C-terminal diglycine motif of ubiquitin characterizing aggresomes [29,33]. This domain, recently solved by X-ray, alone and in complex with ubiquitin, is formed by a compact structure of 5 anti-parallel β-strands, 2 α-helices, and 3 zinc ions with a distinct aromatic pocket [29]. Such a three-dimensional organization is similar to other human zinc finger domains recognizing ubiquitin [34,35]. Ubiquitin interacts with HDAC6 mainly through a hydrogen bond network. The last three residues of ubiquitin are found in an extended conformation which is stabilized by interactions with the HDAC6 aromatic pocket. Arg 1155 and Tyr 1156 residues act as gatekeepers, moving the ubiquitin binding site from an open to a closed conformation [29]. While the mechanism of aggregate recruitment by HDAC6 via ubiquitin is known from a biological [19] and structural [29] point of view, crystallographic information about the two catalytic domains is still missing. The lack of such information is quite problematic for the conception of isoform selective compounds able to modulate HDAC6 activity. Nowadays, this issue is overcome through the generation and refinement of three-dimensional HDAC6 homology models combined with computational interaction and molecular dynamics calculations. Whereas the design of HDAC6 inducers has never been the object of scientific studies, recent reviews accurately describe the structural features that may be interesting for the design of selective HDAC6 inhibitors [22,26,36].

Computational and *in vitro* results were put together to investigate the structural origin of selectivity of the HDAC6 specific inhibitor tubacin [37]. In particular, docking and molecular dynamics calculations highlighted differences in the shape of HDAC surfaces surrounding the binding site. Moreover, the relatively high flexibility of the HDAC6 pocket allowed protein conformational changes by accommodating the cap portion of the studied ligands. These findings were also confirmed by Charrier et al. [38] and by Kozikowski et al. [39], through *in vitro* and docking studies of a set of phenylisoxazole-containing hydroxamates showing IC_50_ values as low as 2 pM for HDAC6. In both studies, the design and modeling of specific inhibitors were based on the differences found in the region adjacent to the HDAC6 catalytic channel, the so-called cap domain. The HDAC6 homology model built by Butler et al. [40] revealed that, while the active site is highly conserved among HDACs, the cap domain differs greatly in terms of shape and properties. Moreover, the rim of the catalytic channel appears wider and shallower in HDAC6 compared to the other HDAC channels. Thus, compounds with bulkier and shorter aromatic moieties were designed. For instance, HDAC6 selectivity was enhanced by adding a large and rigid cap group, such as in tubastatin A [40]. Accordingly, *in vitro* and docking studies on homology models confirmed the HDAC6 selectivity of a series of pyridylalanine-containing hydroxamic acid derivatives [41]. Recently, Kong et al. [42] developed a fluorescent HDAC6 inhibitor characterized by a planar ring of a dansyl moiety interacting with the hydrophobic portion of the HDAC6 cap domain. The molecule also showed an HDAC4 trapping action by sequestrating this specific nuclear isoform in the cytoplasm, which led to a modification of its expression and function.

Another structural selective element of the HDAC6 catalytic site was identified by studying new HDAC6 hydroxamate inhibitors isolated from a virtual screening of 55,000 molecules [43]. The selectivity of these compounds was explained by the presence of a small sub-pocket close to the zinc ion, able to stabilize the position of the thiazole and pyridine rings characterizing such compounds. Moreover, the carbamated form of one of them was shown to act as a prodrug in cell cultures [43].

Table 1 summarizes the key amino acid residues in the second catalytic domain of HDAC6, which are responsible for recognition and binding of inhibitors and ubiquitin [44,45]. The chemical structures of the main HDAC6 specific inhibitors are depicted in Additional file 1.

**Table 1:** Key amino acid residues present in the HDAC6 catalytic pocket and in the binding domain

**HDAC6 catalytic pocket (CDII)**			**HDAC6 ubiquitin binding domain (ZnFUBP)**	
Inhibitor stabilization	Catalytic channel rim (cap domain)	His 499	Ubiquitin stabilization	
		Pro 501		
		Leu 749		
		Phe 679		
		Asp 567		
		Ser 498		
		His 500		
		Glu 502		Arg 1155 **
		Val 503		Tyr 1156 **
		Phe 566		Tyr 1184
		Met 682		Trp 1143
	Internal cavity ZBG + linker domain	Zinc		Tyr 1189
		Asp 649*		Trp 1182
		Asp 742*		
		His 610		
		His 611		
		Pro 608		
		Phe 620		
		Cys 621		
		Tyr 782		
		Phe 680^+^		
		Phe 620^+^		

* zinc coordination; ** gatekeeper; ^+^ hydrophobic sub-pocket.

## HDACs other than HDAC6 act mainly as epigenetic modulators in cognition and neuronal death

Several studies show the implication of HDACs, especially those of class I, in memory processes in mice [9,46]. These processes seem to rely at least in part on epigenetic modulation through HDAC activity on histones, which is demonstrated by the relationship established between cognition, HDAC inhibition and histone acetylation levels [9]. As a matter of fact, HDAC pan-inhibitors were shown to significantly improve long-term memory and learning after inducing neuronal loss in mice. These findings could be correlated with the increase of the acetylation of histones H3 and H4 in the hippocampus and the cortex of mice few hours after being treated [47,48]. Other studies corroborate the influence of histone acetylation levels in memory. One of them showed that the specific modification of acetylation in histone H4 lysine 12 (H4K12) was able to modify the expression of several genes from the hippocampus involved in memory consolidation. The use of the HDAC inhibitor vorinostat (SAHA) was shown to promote the expression of these genes by increasing the acetylation of H4K12, thus resulting in improved cognitive function in mice [49]. Other pan-inhibitors were extensively studied for their effects on *in vitro* and *in vivo* neurodegenerative models. These results, together with the IC_50_ for various HDAC isoforms, are summarized in supplementary material for SAHA (Additional file 2), scriptaid (Additional file 3), trichostatin A (TSA, Additional file 4), sodium butyrate (Additional file 5) and valproic acid (VA, Additional file 6).

The specific roles of the HDAC isoforms have not been completely elucidated so far. The identification of the isoforms playing a major role in memory and neurodegenerative processes would be rather useful in finding specific inhibitors with a therapeutic potential. Up to now, four HDAC isoforms seem to be closely involved in memory processes: HDACs 1 to 4 [5,8,50-60].

Kim et al. [51] showed that HDAC1 was inactivated in CK-p25 mice. These animals are used in models of NDs, since they overexpress protein p25, which has been associated with some AD features: it accumulates in neurons of AD patients and complexes with Cdk5 kinase. This complex is considered responsible for tau hyperphosphorylation and then for cytoskeletal disruption. By inhibiting HDAC1, p25 may lead to important DNA damage and aberrant cell cycle activity, which would contribute to neuronal death [50]. Interestingly, the overexpression of HDAC1, but not HDAC2, in cells and in an *in vivo* model of stroke was able to protect neurons from p25 toxicity [51]. Even if the epigenetic role of nuclear HDAC1 has mostly been associated with neuroprotection, cytosolic HDAC1 was found in damaged axons in the brain of humans suffering from multiple sclerosis, in mouse brain after induced demyelination, as well as in cultured neurons exposed to glutamate and TNF-α [61]. It was then demonstrated that the export of HDAC1 from the nucleus to the cytoplasm was induced by pathological conditions and was essential for the onset of axonal damage. The interaction with the nuclear receptor CRM-1 triggered the nuclear export of HDAC1 that formed complexes with proteins of the kinesine family, finally impairing mitochondrial transport. These events were prevented by the treatment with leuptomycin B, an inhibitor of HDAC1 nuclear export, by non-specific HDAC inhibitors and also by silencing HDAC1 but no other HDACs [61].

The role of HDAC1 and 2 was investigated in primary dissociated hippocampal neurons isolated from floxed HDAC1-, floxed HDAC2-, and floxed HDAC1&2-mice. The deletion of both HDAC1 and 2 during early synaptic development caused a facilitation of excitatory synapse maturation and a modest increase in synapse numbers, which were not observed in the specific deletion of HDAC1 or 2 alone. In contrast, in mature neurons, a decrease of HDAC2 levels alone attenuated basal excitatory transmission without changing the number of detectable nerve terminals. Accordingly, HDAC2-overexpressing mature neurons increased excitatory synapses, suggesting a role for HDAC2 in spontaneous excitatory neurotransmission at least in mature neurons [60].

While the observation of mature hippocampal neurons from mice overexpressing HDAC2 showed an increase in excitatory synapses [60], HDAC2 negatively regulated memory processes and synaptic plasticity after neuron-specific overexpression of HDAC2 in transgenic mice [52]. Moreover, the treatment of these HDAC2-overexpressing mice with the non-specific HDAC inhibitor SAHA resulted in improved memory and increased synapse formation [52].

HDAC3 seems to negatively influence long-term memory processes according to two main observations: first, genetically modified mice with homozygous deletions of *Hdac3* showed improved long-term memory; secondly, the administration of the specific HDAC3 inhibitor RGFP136 also provided memory improvement [54]. In addition to this, the overexpression of HDAC3 resulted in cytotoxicity in cortical neurons and in hippocampally derived HT22 cells, but not in primary kidney fibroblasts, HEK293 and HeLa cell lines [55].

Based on all these published results on nuclear enzymes HDACs 1 to 3, it seems clear that class I HDACs interact with cognitive processes mainly through epigenetic modulation. All of these mechanisms are probably involved together in the enhancement of long-term memory observed *in vivo* after treatment with an HDAC pan-inhibitor (TSA) and a class I specific inhibitor (MS275) [53].

Finally, HDAC4 was also taken into consideration by several authors as a promising target to fight against neurodegeneration. This enzyme, as others from the same class, can shuttle between the nucleus and the cytoplasm [5,8,56]. It was suggested that HDAC4 is a key effector in the multi-step pathway regulating neuronal death. This hypothesis was supported by two major observations: 1) trafficking of HDAC4 from the cytoplasm into the nucleus induced apoptosis in neurons; 2) the inactivation of HDAC4 resulted in protection from neuronal death [57]. In terms of cognition, the intracellular trafficking of HDAC4 was also related to long-term memory in a *Caenorhabditis elegans* model. In this worm, the deletion of *hda4*, a homolog of *hdac4*, resulted in enhanced learning and long-term memory. Moreover, the expression of the mammalian HDAC4 in the neuronal nuclei of the worm was able to impair neuronal function, but not in the cytosol, suggesting that HDAC4 could impair memory formation through inhibition of gene transcription [59].

## The specific role of HDAC6 in the neurodegenerative cascades

There is enough evidence about the involvement of HDAC6 in several NDs and many inferences could be addressed on the basis of results obtained with specific HDAC6 inhibitors. One of the most studied HDAC6 specific inhibitor is tubacin. IC_50_, *in silico* information and biological activities related to NDs are summarized in Table 2 for tubacin together with other known HDAC6 specific inhibitors.

**Table 2:** ***In vitro*activity and*in silico*data of the main HDAC6 specific inhibitors**

											
**Tubacin**											
	**Inhibition of HDAC isoforms**										
	**Class I**				**Class II**						**Class IV**
	**HDAC1**	**HDAC2**	**HDAC3**	**HDAC8**	**HDAC4**	**HDAC5**	**HDAC7**	**HDAC9**	**HDAC6**	**HDAC10**	**HDAC11**
**IC**_**50**_**(nM)**	1400 [40]	6270 [40]	1270 [40]	1270 [40]	17300 [40]	3350 [40]	9700 [40]	4310 [40]	4 [40]	-	3790 [40]
	995 [62]	-	-	6100 [62]	-	-	-	-	28 [62]	-	-
***In silico*data**	Homology modeling, molecular docking and molecular dynamics simulations highlight differences between HDAC1, HDAC6 and HDAC8 [37]										
***In vitro***	**Model**					**Outcomes**			**Observed in**		
	**AD**					Decrease of tau phosphorylation with no disruption of HDAC6-tau interaction [32]			Human embryonic kidney cells (HEK) and HEK cells stably expressing tau (HEK-tau) [32]		
	**PD**					Block of the centrosomal recruitment of parkin [63]			HEK-293T and SH-SY5Y cells [63]		
						Block of the formation of aggresome-like bodies and interference with autophagy [64]			Rat pheochromocytoma cell line (PC12) and SH-SY5Ys [64]		
	**HD**					Neuroprotection [65]			Mouse striatal cells derived from WT htt mice and from *HdhQ*^*109*^ knock-in mice, HEK-293 cells, Cos7 cells, primary cortical neurons [65]		
	**ND and Co**					Disruption of autophagic degradation of aggregated huntingtin [66]			Neuro2a [66]		
						Neuroprotection against oxidative stress [67]			LNCaP, Du145, PC3 HFS and LAPC4 cells [67]		
						Improvement of mitochondrial movement [68]			Rat hippocampal neurons [68]		

**Mercaptoacetamide derivative**											
	**Inhibition of HDAC isoforms**										
	**Class I**				**Class II**						**Class IV**
	**HDAC1**	**HDAC2**	**HDAC3**	**HDAC8**	**HDAC4**	**HDAC5**	**HDAC7**	**HDAC9**	**HDAC6**	**HDAC10**	**HDAC11**
**IC**_**50**_**(nM)**	3220 [69]	7380 [69]	-	-	-	-	-	-	95 [69]	10700 [69]	-
***In vitro***	**Model**					**Outcomes**			**Observed in**		
	**ND and Co**					Neuroprotection against oxidative stress [69]			Rat cortical neurons [69]		

**Tubastatin A**											
	**Inhibition of HDAC isoforms**										
	**Class I**				**Class II**						**Class IV**
	**HDAC1**	**HDAC2**	**HDAC3**	**HDAC8**	**HDAC4**	**HDAC5**	**HDAC7**	**HDAC9**	**HDAC6**	**HDAC10**	**HDAC11**
**IC**_**50**_**(nM)**	16400 [40]	>30000 [40]	>30000 [40]	8540 [40]	>30000 [40]	>30000 [40]	>30000 [40]	>30000 [40]	15 [40]	>30000 [40]	>30000 [40]
***In silico*data**	Homology modeling and molecular docking highlight differences between HDAC1 and HDAC6 [40]										
***In vitro***	**Model**					**Outcomes**			**Observed in**		
	**ND and Co**					Neuroprotection against oxidative stress [40]			Rat primary cortical neurons [40]		
**M344**											
	**Inhibition of HDAC isoforms**										
	**Class I**				**Class II**						**Class IV**
	**HDAC1**	**HDAC2**	**HDAC3**	**HDAC8**	**HDAC4**	**HDAC5**	**HDAC7**	**HDAC9**	**HDAC6**	**HDAC10**	**HDAC11**
**IC**_**50**_**(nM)**	249 [70]	-	-	-	-	-	-	-	**88**[70]	-	-
***In vitro***	**Model**					**Outcomes**			**Observed in**		
	**AD**					Effect on Aβ pathology [71]			Human neuroblastoma cells; rat hippocampal neurons, primary astrocytes, cerebral cortices and midbrain [71]		

**WT-161**											
***In vitro***	**Model**					**Outcomes**			**Observed in**		
	**Myeloma cells**					Increased acetylated α-tubulin (K40) over total acetylated lysine at 2 μM			Human MM1.S cells		
***In vivo***	**Model**					**Outcomes**			**Observed in**		
	**Co**					Increased acetylated α-tubulin (K40) [52]			Area CA1 of hippocampus from mice treated with WT-161 at 25 mg/kg i.p. during 10 days [52]		
						Did not improve cognition [52]			Memory test in mice treated with WT-161 at 25 mg/kg i.p. during 10 days [52]		

The role of HDAC6 as a tubuline deacetylase was investigated within the cascades of various NDs. It is now clear that HDAC6 plays a central role in protein aggregate elimination, in neuronal oxidative stress and in the mitochondrial transport. The implication of HDAC6 in these three particular processes is discussed below.

### HDAC6 and protein aggregates

**Figure 3:**
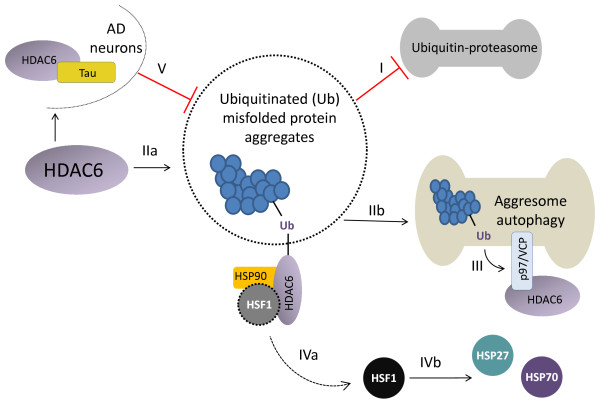
**The role of HDAC6 in various processes related to neurodegeneration.** I) The ubiquitin-proteasome system is impaired in many NDs resulting in the accumulation of highly ubiquitinated misfolded proteins tending to aggregate. II) HDAC6 binds to ubiquitinated protein aggregates (IIa) to constitute a novel organelle, the aggresome (IIb), where they are eventually eliminated by autophagy. III) The AAATPase p97/VCP is able to dissociate the complexes formed between HDAC6 and polyubiquitinated proteins to favor protein degradation. IV) Binding of HDAC6 to polyubiquitinated proteins triggers the dissociation of the HDAC6/HSP90/HSF1 complex, resulting in the activation of HSF1 (IVa). This induces gene expression of HSP70 and HSP27 (IVb), which exert a protective role against the toxic effects of the aggregates in cells. V) In AD neurons, HDAC6 interacts with tau and the excess of tau inhibits the deacetylase and ubiquitin ligase activities of HDAC6.

Protein folding inside cells is not always a spontaneous event. Many synthesized proteins need a complex biological machinery in the cell to achieve their efficient conformation. Large proteins may refold inefficiently, resulting in misfolded intermediates that tend to aggregate [72]. Protein aggregates are usually deleterious to the cell and are a common feature in NDs. HDAC6 is involved within 3 cellular mechanisms able to countervail the accumulation of protein aggregates: 1) the binding to ubiquitinated misfolded proteins, 2) the formation of an aggresome followed by autophagy, and 3) the induction of heat shock proteins (HSPs) (Figure 3). Such mechanisms are involved in the cell response to cytotoxic protein aggregate formation in various NDs [73-75]. The process of autophagy is especially important to counter-balance the accumulation of aggregates since the main route of protein degradation via the ubiquitin-proteasome system is impaired in NDs. Ubiquitin acts as a molecular marker by addressing the substrates to the proteasome 26S in order to be eliminated [76,77]. The accumulation of protein aggregates in NDs may inhibit proteasome activity [78]. This results in an increased number of highly ubiquitinated misfolded proteins commonly observed in AD, PD and HD [78-87]. At this level, HDAC6 was able to increase cell viability under misfolded protein stress [88]. The enzyme could bind polyubiquitinated proteins thanks to its zinc-finger containing domain [89]. This binding led to the active transport of highly ubiquitinated protein aggregates dispersed within the cytoplasm to constitute a novel organelle, the aggresome, where they were eventually eliminated by autophagy [88]. Moreover, HDAC6 interacted with p97/VCP, an AAATPase (ATPase associated with a variety of activities) which is directly involved in protein degradation via the ubiquitin-proteasome system [4,90,91]. As a matter of fact, p97/VCP was able to dissociate the complexes formed between HDAC6 and polyubiquitinated proteins to favor protein degradation. The fate of the ubiquitinated proteins, whether they were being degradated or accumulated into the aggresome depended, at least in part, on the balance between HDAC6 and p97/VCP [92,93].

HDAC6 could also interact with FAT10, a ubiquitin-like modifier which is another marker for protein elimination. In case of ubiquitin-proteasome impairement, HDAC6 promoted FAT10-containing aggresome in order to eliminate marked protein aggregates [94]. The binding of HDAC6 to polyubiquitinated proteins was also shown to trigger the dissociation of the HDAC6/HSP90/HSF1 complex. This led to the activation of heat shock transcription factor 1 (HSF1) which then induced gene expression of chaperones HSP70 and HSP27, exerting a protective role against the toxic effects of the aggregates in cells [4]. On the other hand, HSP90 together with other proteins are involved in tau burden. The loss or inhibition of HDAC6 correlated with a decrease of tau burden in cells [95]. This may be due to increased HSP90 acetylation, which favors degradation of HSP90 client proteins, such as tau. Chaperones, mainly HSP40 and HSP70, also prevented misfolded protein formation [72].

### HDAC6 and oxidative stress

HDAC6 is involved in the deacetylation of peroxiredoxin-1 and peroxiredoxin-2, two enzymes allowing the reduction of peroxides. Since their acetylation increased their reducing properties, HDAC6 inhibition by tubacin could enhance antioxidant properties in the cell [67,96], a mechanism to be considered in the case of neurodegeneration. Moreover this inhibition was able to promote neurite growth on myelin-associated glycoprotein and chondroitin sulfate proteoglycan substrates. TSA, scriptaid and sodium butyrate showed the same effects than tubacin, but their reduced selectivity induced some toxicity [97]. Butler et al. [35] designed a new HDAC6 selective inhibitor, tubastatin A, which showed a neuroprotective effect against oxidative stress induced by homocystic acid. It is also important that no neurotoxicity was observed at the tested concentrations, which is not the case for TSA or other HDAC pan-inhibitors [69,98]. Thus, selectivity seems to be important to avoid some toxicity [40].

### HDAC6 and the mitochondrial transport

An interesting relationship between HDAC6 and mitochondrial transport was established in hippocampal neurons. Microtubule-dependent intracellular trafficking was shown to be regulated by the activity of HDAC6, via the Akt-GSK3-β signaling pathway [68]. As a matter of fact, the inhibition of GSK3-β increased microtubules acetylation and improved at the same time the mitochondrial transport [99]. However, GSK3-β remains a controversial target in NDs since this protein kinase is involved in many biological processes [99]. GSK3-β is present in large quantities in the brain and HDAC6 is one of its targets [68]. The treatment of hippocampal neurons with tubacin, a specific HDAC6 inhibitor, strongly enhanced mitochondrial movement. This inhibition resulted in higher levels of acetylated tubulin and enhanced binding of the motor protein kinesin-1 to tubulin, which promoted the transport of cargo proteins along microtubules [68].

## The role of HDAC6 in Alzheimer’s disease

The main areas of the brain affected by AD are the hippocampus, entorhinal cortex, associative cortex, and amygdala [100]. Neurofibrillary tangles, a typical intraneuronal lesion in AD, follows a defined progression. Gradually, brain regions affected by the accumulation of tau protein expand, starting at the entorhinal region, an adjacent area of the hippocampus. Then, tau pathology reaches the hippocampus, which plays a major role in memory formation (memory training) and more specifically in the memory of events, called declarative or explicit memory [101,102]. The expression of HDAC6 in the hippocampus is thus worth of notice. HDAC6 protein level was increased by 52% in AD cortex and by 91% in AD hippocampus, when compared with young normal brains. In order to confirm that HDAC6 protein is overexpressed in AD, the HDAC6 protein level in the brain of patients with AD was compared to age-matched normal brains.

Proteasome inhibition is a well documented feature in AD and seems to potentiate HDAC6-tau interaction. Such interaction could be observed *in vitro*, in cells and in brains of AD patients. HDAC6 and tau co-localised within the perinuclear aggresome-like compartment, independently of the tubulin deacetylase activity of HDAC6. Treatment with tubacin or HDAC6 knockdown *in vivo* did not impair the interaction between HDAC6 and tau but was able to decrease tau phosphorylation [32]. Moreover, the post-mortem study of the brain of AD patients denoted low levels of α-tubulin and increased levels of tubulin acetylation. These events were mainly observed in neurons presenting neurofibrillary tangles. By binding to HDAC6, tau inhibited the deacetylase activity and led to an increase in tubulin acetylation. This increase was also observed in human cells over-expressing tau protein. The excess of tau acted as an HDAC6 inhibitor and prevented the autophagy induction followed by proteasome inhibition in cells. Thus, tau can act as an inhibitor of both the deacetylase activity of HDAC6 and the aggresome pathway, depending upon the HDAC6 binding to polyubiquitinated proteins [103]. Ding et al. [32] hypothesized that even if HDAC6 up-regulation in AD brains contributes to the sequestration of ubiquitinated protein aggregates and recruitment of autophagic components, it would eventually be deleterious to cell survival in AD, due to decreased tubulin acetylation and increased tau phosphorylation. Finally, the positive impact of HDAC6 depletion in cognition has been recently demonstrated by crossing HDAC6 knockdown mice with a model for severe amyloid pathology. The loss of HDAC6 improved the impairment of associative and spatial memory formation and was able to recover the deficits in mitochondrial trafficking induced by Aβ [104]. These findings suggest HDAC6 inhibition as a promising target in AD.

## The role of HDAC6 in Parkinson’s disease

PD affects the extrapyramidal system and is characterized by a progressive degeneration of the nigrostriatal dopaminergic (DA) pathway with the presence of Lewy bodies (LBs), which are cytoplasmic inclusions mainly composed of α-synuclein. During the disease, α-synuclein accumulates in an insoluble form within the substantia nigra pars compacta (where HDAC6 is expressed, Figure 1) and other regions [105]. HDAC6 is an attractive target in this process, since this isoform was involved in the elimination of protein aggregates of α-synuclein within a cellular model of PD [64]. Most of the LBs are located in the nigrostriatal DA system but other systems may be affected, including the locus coeruleus (where HDAC6 is also well expressed, Figure 1) and the nucleus basalis of Meynert [106]. HDAC6 was also found in the cortex (Figure 1), another region affected in PD by the presence of LBs and neurofibrillary tangles.

There are some uncertainties on the specific role of LBs in PD [107,108]. While several authors claimed the *in vitro* cytotoxicity of α-synuclein in DA neurons [84,85,105,109], Du et al. [110] demonstrated in the *Drosophila* model of PD that LBs-like inclusions were rather cytoprotective in the process of PD neurodegeneration. Here, the crucial role of HDAC6 is highlighted. HDAC6 promoted the formation of inclusions from α-synuclein toxic oligomers. The amount of inclusions was increased when HDAC6 was coexpressed with α-synuclein in DA neurons, compared to neurons expressing only α-synuclein. Thus, HDAC6 exerted a cytoprotective role in DA neurons by allowing inclusion formation and decreasing the amount of α-synuclein oligomers in the fly PD model [110]. Furthermore, HDAC6 co-localized with α-synulcein in the perinuclear region to form aggresome-like bodies in a MPP^+^ induced cellular model of PD. In this specific case, HDAC6 knockdown or inhibition by tubacin resulted not only in the reduction of perinuclear inclusions but also in the increase of α-synuclein in the nucleus with increased cell death. The nuclear accumulation of α-synuclein after HDAC6 inhibition may be strongly related to the role of HDAC6 in the autophagic process of misfolded proteins as an elimination route, an alternative to the ubiquitin-proteasome system impaired in PD and other NDs [64]. A mutation in the gene encoding DJ-1 resulted in misfolding and accumulation of this protein, which is characteristic of an early-onset form of PD. Misfolded DJ-1 aggregates were eliminated by autophagy via parkin-HDAC6 binding [97]. Parkin is an E3 ligase that participates in the addition of ubiquitin molecules in order to mark misfolded proteins. A mutation in the gene encoding parkin is also responsible for an autosomal-recessive form of early PD [111]. In case of proteasome impairment, parkin formed a complex with the heterodimeric E2 enzyme UbcH13/Uev1a to polyubiquitinate misfolded DJ-1 [97]. Even if the mechanisms by which parkin suppressed parkinsonism remain unclear, parkin participated in the elimination of impaired mitochondria (mitophagy) via the HDAC6-dependent mitochondria ubiquitination [112]. Moreover, the HDAC6 inhibitor tubacin prevented the recruitment of parkin by the centrosome via proteasome inhibition [63].

## The role of HDAC6 in Huntington’s disease

HD is caused by gene modification of CAG trinucleotide repeats expansion, resulting in pathological polyglutamine expansion in proteins, and leading to the accumulation of huntingtin aggregates [86,113-115]. The toxicity of these aggregates in a *Drosophila* model correlated with changes in histone acetylation, and transcriptional failing [80]. In a transgenic mouse model of HD, the expression of many genes was considerably modified in the striatum. Some components of the ubiquitin-proteasome had their expression increased. This is interesting in the context of this disease, since the ubiquitin-proteasome is directly related to the accumulation of huntingtin [116].

In a *Drosophila* model of HD, the co-expression of human chaperones HSP70 and HSP40 acted synergistically to suppress the degenerative phenotype without changing the morphology of cell huntingtin aggregates. In addition, HSP70 and HSP40 were sequestered within the cell aggregates of huntingtin. This might significantly reduce the intracellular availability of these chaperones and lead to an increase in abnormal folding of proteins [117]. Thus, the presence of HDAC6 in these structures may have a protective role in HD through its capacity to induce protective chaperones (cf. chapter 4) [4].

HDAC6 was shown to be expressed in both structures of the striatum, caudate nucleus and putamen (Figure 1), where the most outstanding neuropathological process of HD takes place.

It has been suggested that HDAC6 forms a complex with both dynein and aggregates (via ubiquitin binding), linking protein aggregates to the microtubule motor necessary for the autophagic process. HDAC6 deacetylase activity was shown to be important for this whole process in HD cell models, since the specific inhbition of HDAC6 deacetylase activity blocked the recruitment of components of the autophagy machinery to the aggresome. The yet non-elucidated mechanism by which the HDAC6 deacetylase activity is essential to autophagy may be linked to the acetylation levels of HDAC6 substrates [66]. Taken together, these data suggest that an inhibition would not be beneficial for the elimination of protein aggregates in HD. However, the inhibition of HDAC6 stimulated the microtubule-dependent trafficking of vesicles, a process which appears deficient in HD. The inhibition of the deacetylase activity of HDAC6 by TSA and SAHA increased tubulin acetylation and resulted in an improved intracellular vesicle trafficking and release of BDNF. These two processes are disrupted in neurons affected by HD. Even if TSA and SAHA are not specific inhibitors of HDAC6, the authors of this study attributed the observed effects to HDAC6 inhibition based on the fact that MS-275, a specific inhibitor of HDAC1, had no effect on the release of BDNF [65]. Despite these promising results, a study conducted with Hdac6 knockout mice failed to prove the influence of HDAC6 genetic depletion to the clinical manifestations of HD. Even if acetylation levels of tubulin were increased in this model, no effect could be observed in the disease progression. Accordingly, the transport of BDNF from the cortex to the striatum was not improved in this model [118].

## The role of HDAC6 in other neurodegenerative diseases

HDAC6 may also be implicated in several other NDs including FTLD, ALS and CMT [119,120]. In FTLD and ALS, the down regulation of HDAC6 was observed in a *Drosophila* model based on the knockout of transactive response DNA-binding protein (TDP-43). Inclusions of TDP-43 are hallmarks of FTDL and ALS, and the silencing of TDP-43 in cells caused HDAC6 downregulation which increased proteotoxicity [120]. On the opposite, in CMT, HDAC6 inhibition could be an interesting therapeutic strategy. Symptomatic improvement was observed in a transgenic mouse model of CMT after the treatment with specific HDAC6 inhibitors, together with the increase in tubulin acetylation [119].

## Critical insights on HDAC6 as a target to fight against neurodegeneration

There is no doubt that HDAC6 is involved in several events of the neurodegenerative cascades and differs from other HDACs not only from a structural point of view, but also in its subcellular localization. Impaired mitochondrial transport and elimination of protein aggregates are common features in various NDs and are linked to both deacetylase and ubiquitin ligase activities of HDAC6. However, it seems that the results obtained with the specific inhibition of HDAC6 in neurodegenerative models cannot be extrapolated from one disease to another. One possible explanation is that different diseases involve specific proteins and the protein-protein interactions (PPI) are not to be neglected in the case of HDAC6. One example is the interaction between HDAC6 and tau, leading to the inhibition of HDAC6 and preventing its role in autophagy [32]. In the case of NDs, PPI mechanisms should be further studied not only in the case of HDAC6, but also in the case of class I HDACs. As a matter of fact, class I HDAC inhibition succeeded to improve cognition, memory and learning in animal models, depending on the disease and the targeted isoform (Additional files). Considering HDAC1 and 2, despite the high level of identity between these isoforms, various outcomes were observed through the overexpression of one enzyme or the other. This may be related to the role of PPI in HDAC activity modulation. In this way, HDAC1 may play a role in neurodegeneration not only via epigenetic mechanisms as HDAC2, but also upon nuclear export followed by its interaction with the nuclear factor CRM-1 and kinesin complex formation in order to intervene with mitochondrial transport [61].

Even if the specific inhibition of HDAC6 would be beneficial to countervail mitochondrial transport impairement and oxidative stress in NDs [67,68,96], as well as tau accumulation [95] in general cell assays, the role of HDAC6 in protein aggregate elimination was also highlighted [88]. Moreover, WT-161, a selective HDAC6 inhibitor failed to improve cognitive function in a mouse memory test [49]. The *in vitro* and *in vivo* results of a specific HDAC6 inhibition (Table 2) did not show an improvement of the cognitive function in NDs. Also, there was a strong evidence that HDAC6 was inhibited by tau in AD [103]. Recently, cognitive improvement was obtained in a mouse model of AD crossed with HDAC6 knockout. Thus, while HDAC6 seems to be necessary for aggregate elimination by autophagy, an induction could also fail to countervail AD pathological conditions, since overexpressed HDAC6 would be eventually inhibited by tau (Figure 3). In this regard, it would be interesting to further investigate PPI between HDAC6 and other proteins involved in specific neurodegenerative processes. PPI inhibitors have been of great interest in drug discovery since they allowed to interact with the specific pathway of an enzyme without interfering with the enzyme activity needed for other processes. Moreover, PPI could be also modulated by small molecules [121], which is a requested feature in order to allow oral administration and blood–brain barrier permeability. Thus, the study of PPI underlying HDAC6 mechanisms seems to be a promising approach in modulating HDAC6 activity in the context of neurodegeneration.

## Abbreviations

AAATPase: ATPase associated with a variety of activities; AD: Alzheimer’s disease; ALS: Amyotrophic lateral sclerosis; Akt: Protein kinase B (also known as PKB); Aβ: Amyloid β-protein; BDNF: Brain-derived neurotrophic factor; Cdk5: Cyclin-dependent kinase-5; CMT: Charcot-Marie-Tooth disease; Co: Cognition; CRM-1: Chromosome region maintenance (nuclear receptor); DA: Dopaminergic; DJ-1: Parkinson disease (autosomal recessive, early onset) 7 (also known as PARK7); FTDL: Frontotemporal dementia; GSK3-β: Glycogen synthase kinase 3-β; HAT: Histone acetyltransferase; HD: Huntington’s disease; HDAC: Histone deacetylase; HDLP: Histone deacetylase-like protein; HSF: Heat shock factor protein; HSP: Heat shock protein; LBs: Lewy bodies; MEF2: Myocyte enhancing factor-2; NDs: Neurodegenerative diseases; PD: Parkinson’s disease; PPI: Protein-protein interaction; SAGE: Serial analysis of gene expression; SAHA: Suberoylanilide hydroxamic acid, vorinostat; TNF-α: Tumor necrosis factor-α; TSA: Trichostatin A; UbcH13: Ubiquitin conjugated enzyme; Uev1a: Ubiquitin conjugated enzyme variant; VA: Valproic acid; VCP: Valosin-containing protein.

## Misc

Claudia Simões-Pires, Vincent Zwick, contributed equally.

## Competing interests

Dr Esther Schenker is an employee of Les Laboratoires Servier, France.

## Authors’ contributions

CSP designed the first draft with the main ideas to be discussed in this manuscript. CSP and VZ reviewed the literature and drafted the whole manuscript, except for the structural part that was performed by AN. ES gave valuable information in the field of epigenetics and helped to draft the manuscript. PAC and MC coordinated the work and helped to draft the manuscript. All authors read and approved the final manuscript.

## Supplementary Material

Additional file 1 HDAC6 specific inhibitors. Click here for file

Additional file 2 Activity of vorinostat on HDACs. Click here for file

Additional file 3 Activity of scriptaid on HDACs. Click here for file

Additional file 4 Activity of trichostatin A on HDACs. Click here for file

Additional file 5 Activity of sodium butyrate on HDACs. Click here for file

Additional file 6 Activity of valproic acid on HDACs. Click here for file
