# Supplementary material for: HDAC6 as a target for neurodegenerative diseases: what makes it different from the other HDACs?
Source: Mol Neurodegener. 2013 Jan 29;8:7. doi: 10.1186/1750-1326-8-7 (PMC3615964; doi:10.1186/1750-1326-8-7)
Supplement: Additional file 1 — HDAC6 specific inhibitors. [file 1750-1326-8-7-S1.docx]

Additional file 1. Activity of vorinostat on HDACs.

|  |  | **Inhibition of HDAC isoforms** | | | | | | | | | | | | | |
| --- | --- | --- | --- | --- | --- | --- | --- | --- | --- | --- | --- | --- | --- | --- | --- |
| **Vorinostat (SAHA)** |  | **HDACs** | **Class I** | | | | | **Class II** | | | | | | | **Class IV** |
|  |  |  | **HDAC1** | | **HDAC2** | **HDAC3** | **HDAC8** | **HDAC4** | **HDAC5** | | **HDAC7** | **HDAC9** | **HDAC6** | **HDAC10** | **HDAC11** |
|  |  | **IC_50_ (nM)** | 68 [1] | | 164 [1] | 48 [1] | 1524 [1] | 101 [1] | >50000 [1] | | 104 [1] | 107 [1] | 90 [1] | - | - |
|  |  |  | 14 [2] | | 62 [2] | 869 [2] | 7 [2] | >50000 [2] | - | | >50000 [2] | >50000 [2] | 5500 [2] | - | - |
|  |  |  | 96 [3] | | 282 [3] | 17 [3] | 1140 [3] | - | - | | - |  | 14 [3] | 72 [3] | - |
|  |  |  | | | | | | | | | | | | | |
|  |  |  | **Disease** | **Outcomes** | | | | | | **Observed in** | | | | | |
|  |  | ***In vitro* outcomes** | **AD** | Effect on Aβ plaque pathology [4,5] | | | | | | Human neuroblastoma cells, rat primary astrocytes, cerebral cortices and midbrain, rat hippocampal neurons [5], human astrocytes [4] | | | | | |
|  |  |  | **PD** | Neuroprotection against toxicity of α-synuclein [6] | | | | | | Transfected SH-SY5Y cells [6] | | | | | |
|  |  |  |  | Neuroprotection against toxicity of MPP^+^ [7] | | | | | | Human derived SK-N-SH and rat derived MES 23.5 cells [7] | | | | | |
|  |  |  | **HD** | Neuroprotection against oxidative stress [8] | | | | | | Cells from rat cerebral cortex [8] | | | | | |
|  |  |  |  | Neuroprotection against polyglutamine toxicity [9] | | | | | | Transfected MN-1 cells expressing mutant polyglutamine [9] | | | | | |
|  |  |  | **ND and Co** | Neuroprotection against oxidative stress [10] | | | | | | LNCaP, Du145, PC3 HFS and LAPC4 cells [10] | | | | | |
|  |  | ***In vivo* outcomes** | **AD** | Improvement of contextual memory [11] | | | | | | Mouse model of AD (APPswe/PS1dE9) [11] | | | | | |
|  |  |  | **PD** | Neuroprotection against toxicity of α-synuclein [6] | | | | | | Transgenic *Drosophila*[6] | | | | | |
|  |  |  | **HD** | Improvement of motor function [12] | | | | | | R6/2 mice [12] | | | | | |
|  |  |  |  | Neuroprotection against polyglutamine toxicity [13] | | | | | | Two *Drosophila* models of polyglutamine disease [13] | | | | | |
|  |  |  | **ND and**  **Co** | Improvement of learning and memory [14,15] | | | | | | *Hdac2*-deficient mice [14]  C57BL/6 mice [15] | | | | | |

**

**

AD: Alzheimer’s disease; PD: Parkinson’s disease; HD: Hungtington’s disease; ND: neurodegeneration; Co: cognition.

Table references

1. Khan N, Jeffers M, Kumar S, Hackett C, Boldog F, Khramtsov N, Qian X, Mills E, Berghs SC, Carey N et al.: **Determination of the class and isoform selectivity of small-molecule histone deacetylase inhibitors.** *Biochem J* 2008, **409:**581-589.

2. Huber K, Doyon G, Plaks J, Fyne E, Mellors JW, Sluis-Cremer N: **Inhibitors of histone deacetylases.** *J Biol Chem* 2011, **286:**22211-22218.

3. Kozikowski AP, Tapadar S, Luchini DN, Kim KH, Billadeau DD: **Use of the nitrile oxide cycloaddition (NOC) reaction for molecular probe generation: a new class of enzyme selective histone ceacetylase inhibitors (HDACIs) showing picomolar activity at HDAC6.** *J Med Chem* 2008, **51:**4370-4373.

4. Nuutinen T, Suuronen T, Kauppinen A, Salminen A: **Valproic acid stimulates clusterin expression in human astrocytes: Implications for Alzheimer's disease.** *Neurosci Lett* 2010, **475:**64-68.

5. Nuutinen T, Suuronen T, Kyrylenko S, Huuskonen J, Salminen A: **Induction of clusterin/apoJ expression by histone deacetylase inhibitors in neural cells.** *Neurochem Int* 2005, **47:**528-538.

6. Kontopoulos E, Parvin JD, Feany MB: **a-synuclein acts in the nucleus to inhibit histone acetylation and promote neurotoxicity.** *Human Mol Gen* 2006, **15:**3012-3023.

7. Kidd SK, Schneider JS: **Protection of dopaminergic cells from MPP(+)-mediated toxicity by histone deacetylase inhibition.** *Brain Res* 2010, **1354:**172-178.

8. Ryu H, Lee J, Olofsson BA, Mwidau A, Deodoglu A, Escudero M, Flemington E, Azizkhan-Clifford J, Ferrante RJ, Ratan RR: **Histone deacetylase inhibitors prevent oxidative neuronal death independent of expanded polyglutamine repeats via an Sp1-dependent pathway.** *Proc Natl Acad Sci U S A* 2003, **100:**4281-4286.

9. McCampbell A, Taye AA, Whitty L, Penney E, Steffan JS, Fischbeck KH: **Histone deacetylase inhibitors reduce polyglutamine toxicity.** *Proc Natl Acad Sci U S A* 2001, **98:**15179-15184.

10. Parmigiani R, Xu W, Venta-Perez G, Erdjument-Bromage H, Yaneva M, Tempst P, Marks P: **HDAC6 is a specific deacetylase of peroxiredoxins and is involved in redox regulation.** *Proc Natl Acad Sci U S A* 2008, **105:**9633-9638.

11. Kilgore M, Miller C, Fass DM, Hennig KM, Haggarty SJ, Sweatt JD, Rumbaugh G: **Inhibitors of class 1 histone deacetylases reverse contextual memory deficits in a mouse model of Alzheimer's disease.** *Neuropsychopharmacol* 2009, **35:**870-880.

12. Hockly E, Richon VM, Woodman B, Smith DL, Zhou XB, Rosa E, Sathasivam K, Ghazi-Noori S, Mahal A, Lowden PAS et al.: **Suberoylanilide hydroxamic acid, a histone deacetylase inhibitor, ameliorates motor deficits in a mouse model of Huntington's disease.** *Proc Natl Acad Sci USA* 2003, **100:**2041-2046.

13. Steffan JS, Bodai L, Pallos J, Poelman M, McCampbell A, Apostol BL, Kazantsev A, Schmidt E, Zhu YZ, Greenwald M et al.: **Histone deacetylase inhibitors arrest polyglutamine-dependent neurodegeneration in Drosophila.** *Nature* 2001, **413:**739-743.

14. Guan JS, Haggarty SJ, Giacometti E, Dannenberg JH, Joseph N, Gao J, Nieland TJF, Zhou Y, Wang X, Mazitschek R et al.: **HDAC2 negatively regulates memory formation and synaptic plasticity.** *Nature* 2009, **459:**55-60.

15. Peleg S, Sananbenesi F, Zovoilis A, Burkhardt S, Bahari-Javan S, Agis-Balboa RC, Cota P, Wittnam JL, Gogol-Doering A, Opitz L et al.: **Altered Histone acetylation is associated with age-dependent memory impairment in mice.** *Science* 2010, **328:**753-756.
