# Supplementary material for: HDAC6 as a target for neurodegenerative diseases: what makes it different from the other HDACs?
Source: Mol Neurodegener. 2013 Jan 29;8:7. doi: 10.1186/1750-1326-8-7 (PMC3615964; doi:10.1186/1750-1326-8-7)
Supplement: Additional file 2 — Activity of vorinostat on HDACs. [file 1750-1326-8-7-S2.docx]

Additional file 2. Activity of scriptaid on HDACs.

| **Scriptaid** |  | **Inhibition of HDAC isoforms** | | | | | | | | | | | | | |
| --- | --- | --- | --- | --- | --- | --- | --- | --- | --- | --- | --- | --- | --- | --- | --- |
|  |  | **HDACs** | **Class I** | | | | | **Class II** | | | | | | | **Class IV** |
|  |  |  | **HDAC1** | **HDAC2** | | **HDAC3** | **HDAC8** | **HDAC4** | **HDAC5** | **HDAC7** | | **HDAC9** | **HDAC6** | **HDAC10** | **HDAC11** |
|  |  | **IC_50_ (nM)** | 0.6 [1] | 1 [1] | | 607 [1] | 14 [1] | 14000 [1] | >50000 [1] | 2200 [1] | | >50000 [1] | 34 [1] | - | - |
|  |  |  | | | | | | | | | | | | | |
|  |  |  | **Disease** | | **Outcomes** | | | | | | **Observed in** | | | | |
|  |  | ***In vitro***  **outcomes** | **AD** | | Effect on Aβ plaque pathology [2] | | | | | | Human astrocytes [2] | | | | |
|  |  |  | **ND and Co** | | Disruption of aggregosome formation [3] | | | | | | COS1 cells [3] | | | | |
|  |  |  |  |  | Neuroprotection against oxidative stress [4-6] | | | | | | Rat cerebral cortex neurons [4,5], rat dorsal root ganglion neurons [6] | | | | |

AD: Alzheimer’s disease; ND: neurodegeneration; Co: cognition.

Table references

1. Huber K, Doyon G, Plaks J, Fyne E, Mellors JW, Sluis-Cremer N: **Inhibitors of histone deacetylases.** *J Biol Chem* 2011, **286:**22211-22218.

2. Nuutinen T, Suuronen T, Kauppinen A, Salminen A: **Valproic acid stimulates clusterin expression in human astrocytes: Implications for Alzheimer's disease.** *Neurosci Lett* 2010, **475:**64-68.

3. Corcoran LJ, Mitchison TJ, Liu Q: **A novel action of histone deacetylase inhibitors in a protein aggresome disease model.** *Curr Biol* 2004, **14:**488-492.

4. Langley B, D'Annibale MA, Suh K, Ayoub I, Tolhurst A, Bastan B, Yang L, Ko B, Fisher M, Cho S et al.: **Pulse inhibition of histone deacetylases induces complete resistance to oxidative death in cortical neurons without toxicity and reveals a role for cytoplasmic p21waf1/cip1 in cell cycle-independent neuroprotection.** *J Neurosci* 2008, **28:**163-176.

5. Kozikowski AP, Chen Y, Gaysin A, Chen B, D'Annibale MA, Suto CM, Langley BC: **Functional differences in epigenetic modulators - superiority of mercaptoacetamide-based histone deacetylase inhibitors relative to hydroxamates in cortical neuron neuroprotection studies.** *J Med Chem* 2007, **50:**3054-3061.

6. Rivieccio MA, Brochier C, Willis DE, Walker BA, D'Annibale MA, McLaughlin K, Siddiq A, Kozikowski AP, Jaffrey SR, Twiss JL et al.: **HDAC6 is a target for protection and regeneration following injury in the nervous system.** *Proc Natl Acad Sci U S A* 2009, **106:**19599-19604.
