# Supplementary material for: HDAC6 as a target for neurodegenerative diseases: what makes it different from the other HDACs?
Source: Mol Neurodegener. 2013 Jan 29;8:7. doi: 10.1186/1750-1326-8-7 (PMC3615964; doi:10.1186/1750-1326-8-7)
Supplement: Additional file 3 — Activity of scriptaid on HDACs. [file 1750-1326-8-7-S3.docx]

|  |  | **Inhibition of HDAC isoforms** | | | | | | | | | | | | |
| --- | --- | --- | --- | --- | --- | --- | --- | --- | --- | --- | --- | --- | --- | --- |
| **Trichostatin A (TSA)** |  | **HDACs** | **Class I** | | | | **Class II** | | | | | | | **Class IV** |
|  |  |  | **HDAC1** | **HDAC2** | **HDAC3** | **HDAC8** | **HDAC4** | **HDAC5** | | **HDAC7** | **HDAC9** | **HDAC6** | **HDAC10** | **HDAC11** |
|  |  | **IC_50_ (nM)** | 2 [1] | 3 [1] | 4 [1] | 456 [1] | 6 [1] | - | | 5 [1] | 6 [1] | 3 [1] | - | - |
|  |  |  | 4 [2] | 14 [2] | 2 [2] | 1 [2] | - | - | | - | - | 1 [2] | 5 [2] | - |
|  |  |  | | | | | | | | | | | | |
|  |  |  | **Disease** | **Outcomes** | | | | | **Observed in** | | | | | |
|  |  | ***In vitro* outcomes** | **AD** | Increase of choline acetyltransferase (CHAT) activity [3] | | | | | Cultured rat sympathetic neurons [3] | | | | | |
|  |  |  |  | Effect of Aβ plaque pathology [4,5] | | | | | Human neuroblastoma cells, rat primary astrocytes, rat cerebral cortices and midbrain, rat hippocampal neurons [5]; Human astrocytes [4] | | | | | |
|  |  |  | **PD** | Neuroprotection against excitotoxicity [6] | | | | | Cerebellar granule cells and rat neuron-enriched cerebral cortical cells [6] | | | | | |
|  |  |  |  | Improvement of toxicity against 1-methyl-4- phenylpyridinium and rotenone [7] | | | | | Dopaminergic neurons [7] | | | | | |
|  |  |  |  | Block of the centrosomal recruitment of parkin when proteasome is inhibited [8] | | | | | HEK293T and SH-SY5Y cells [8] | | | | | |
|  |  |  |  | Up-regulation of GDNF and BDNF expression [9] | | | | | Neuron-glia from F344 rats [9] | | | | | |
|  |  |  |  | Neuroprotection against pro-inflammatory stimuli [10] | | | | | Ventral mesencephalic neuron-glia and microglia from F344 rats [10] | | | | | |
|  |  |  | **HD** | Neuroprotection against oxidative stress [11-14] | | | | | Cells from rat cerebral cortex [11]; *C. elegans* neurons expressing a human huntingtin fragment [12]; PC12 cells transfected with a part of the DRPLA gene [13]; *S. cerevisiae* expressing expanded polyglutamine [14] | | | | | |
|  |  |  |  | Neuroprotection by promoting the intracellular transport of BDNF [15] | | | | | Mouse striatal cells derived from WT htt mice and from *HdhQ^109^* knock-in mice, HEK293 cells, Cos7 cells, primary cortical neurons [15] | | | | | |
|  |  |  |  | Neuroprotection against polyglutamine toxicity [16, 17] | | | | | Transfected MN-1 cells expressing mutant polyglutamine [16] | | | | | |
|  |  |  |  |  | | | | | Transfected HEK293T, Neuro-2a and DU145 cells [17] | | | | | |
|  |  |  | **ND and Co** | Neuroprotection [18-22] | | | | | Rat astrocytes and cerebral cortical neurons [18,19,21,22], cerebellar granule neurons from mice C57BL/6 [20] | | | | | |
|  |  |  |  | Neuroprotection against oxidative stress [23-25] | | | | | Rat cortical neurons [23,25], rat dorsal root ganglion neurons and cortical neurons [24] | | | | | |
|  |  |  |  | Neuroprotection against excitotoxicity [26] | | | | | Rat mature cerebellar granule cells [26] | | | | | |
|  |  |  |  | Apoptosis induction in neuronal cells[27] | | | | | Rat cerebellar granule neurons and murine Neuro-2a neuroblastoma cells [27,27] | | | | | |
|  |  |  |  | Increase of LPS-induced inflammatory response [28] | | | | | Murine N9 microglia and rat primary astrocytes, microglia and hippocampal cells [28] | | | | | |
|  |  | ***In vivo* outcomes** | **AD** | Improvement of learning and memory [29] | | | | | APP/PS1 mice[29], CK-p25 Tg mice [30], Sprague-Dawley rats [31], C57BL/6 mice[32], C57BL/6J mice [33], C57BL/6J mice [34] | | | | | |

Additional file 3. Activity of trichostatin A on HDACs.



AD: Alzheimer’s disease ; PD : Parkinson’s disease; HD: Hungtington’s disease; ND: neurodegeneration; Co: cognition.

Table references

1. Khan N, Jeffers M, Kumar S, Hackett C, Boldog F, Khramtsov N, Qian X, Mills E, Berghs SC, Carey N et al.: **Determination of the class and isoform selectivity of small-molecule histone deacetylase inhibitors.** *Biochem J* 2008, **409:**581-589.

2. Kozikowski AP, Tapadar S, Luchini DN, Kim KH, Billadeau DD: **Use of the nitrile oxide cycloaddition (NOC) reaction for molecular probe generation: a new class of enzyme selective histone ceacetylase inhibitors (HDACIs) showing picomolar activity at HDAC6.** *J Med Chem* 2008, **51:**4370-4373.

3. Chireux M, Espinos E, Bloch S, Yoshida M, Weber MJ: **Histone hyperacetylating agents stimulate promoter activity of human choline acetyltransferase gene in transfection experiment.** *Mol Brain Res* 1996, **39:**68-78.

4. Nuutinen T, Suuronen T, Kauppinen A, Salminen A: **Valproic acid stimulates clusterin expression in human astrocytes: Implications for Alzheimer's disease.** *Neurosci Lett* 2010, **475:**64-68.

5. Nuutinen T, Suuronen T, Kyrylenko S, Huuskonen J, Salminen A: **Induction of clusterin/apoJ expression by histone deacetylase inhibitors in neural cells.** *Neurochem Int* 2005, **47:**528-538.

6. Leng Y, Chuang DM: **Endogenous a-Synuclein is induced by Valproic scid through histone deacetylase inhibition and participates in neuroprotection against glutamate-induced excitotoxicity.** *J Neurosci* 2006, **26:**7502-7512.

7. Wang Y, Wang X, Liu L, Wang X: **HDAC inhibitor trichostatin A-inhibited survival of dopaminergic neuronal cells.** *Neurosci Lett* 2009, **467:**212-216.

8. Jiang Q, Ren Y, Feng J: **Direct binding with histone deacetylase 6 mediates the reversible recruitment of Parkin to the centrosome.** *J Neurosci* 2008, **28:**12993-13002.

9. Wu X, Chen PS, Dallas S, Wilson B, Block ML, Wang CC, Kinyamu H, Lu N, Gao X, Leng Y et al.: **Histone deacetylase inhibitors up-regulate astrocyte GDNF and BDNF gene transcription and protect dopaminergic neurons.** *Int J Neurophsychopharmacol* 2008, **11:**1123-1134.

10. Chen PS, Wang CC, Bortner CD, Peng GS, Wu X, Pang H, Lu RB, Gean PW, Chuang DM, Hong JS: **Valproic acid and other histone deacetylase inhibitors induce microglial apoptosis and attenuate lipopolysaccharide-induced dopaminergic neurotoxicity.** *Neuroscience* 2007, **149:**203-212.

11. Ryu H, Lee J, Olofsson BA, Mwidau A, Deodoglu A, Escudero M, Flemington E, Azizkhan-Clifford J, Ferrante RJ, Ratan RR: **Histone deacetylase inhibitors prevent oxidative neuronal death independent of expanded polyglutamine repeats via an Sp1-dependent pathway.** *Proc Natl Acad Sci U S A* 2003, **100:**4281-4286.

12. Bates EA, Victor M, Jones AK, Shi Y, Hart AC: **Differential contributions of caenorhabditis elegans histone deacetylases to Huntingtin polyglutamine toxicity.** *J Neurosci* 2006, **26:**2830-2838.

13. Kariya S, Hirano M, Uesato S, Nagai Y, Nagaoka Y, Furiya Y, Asai H, Fujikake N, Toda T, Ueno S: **Cytoprotective effect of novel histone deacetylase inhibitors against polyglutamine toxicity.** *Neurosci Lett* 2006, **392:**213-215.

14. Hughes RE, Lo RS, Davis C, Strand AD, Neal CL, Olson JM, Fields S: **Altered transcription in yeast expressing expanded polyglutamine.** *Proc Natl Acad Sci USA* 2001, **98:**13201-13206.

15. Dompierre JP, Godin JD, Charrin BC, Cordelieres FP, King SJ, Humbert S, Saudou F: **Histone deacetylase 6 inhibition compensates for the transport deficit in Huntington's disease by increasing tubulin acetylation.** *J Neurosci* 2007, **27:**3571-3583.

16. McCampbell A, Taye AA, Whitty L, Penney E, Steffan JS, Fischbeck KH: **Histone deacetylase inhibitors reduce polyglutamine toxicity.** *Proc Natl Acad Sci U S A* 2001, **98:**15179-15184.

17. Li Y, Yokota T, Gama V, Yoshida T, Gomez JA, Ishikawa K, Sasaguri H, Cohen HY, Sinclair DA, Mizusawa H et al.: **Bax-inhibiting peptide protects cells from polyglutamine toxicity caused by Ku80 acetylation.** *Cell Death Differ* 2007, **14:**2058-2067.

18. Marinova Z, Leng Y, Leeds P, Chuang DM: **Histone deacetylase inhibition alters histone methylation associated with heat shock protein 70 promoter modifications in astrocytes and neurons.** *Neuropharmacol* 2011, **60:**1109-1115.

19. Marinova Z, Ren M, Wendland JR, Leng Y, Liang MH, Yasuda S, Leeds P, Chuang DM: **Valproic acid induces functional heat-shock protein 70 via class I histone deacetylase inhibition in cortical neurons: a potential role of Sp1 acetylation.** *J Neurochem* 2009, **111:**976-987.

20. Bolger TA, Yao TP: **Intracellular trafficking of Histone Deacetylase 4 regulates neuronal cell death.** *J Neurosci* 2005, **25:**9544-9553.

21. Jeong MR, Hashimoto R, Senatorov VV, Fujimaki K, Ren M, Lee MS, Chuang DM: **Valproic acid, a mood stabilizer and anticonvulsant, protects rat cerebral cortical neurons from spontaneous cell death: a role of histone deacetylase inhibition.** *FEBS Lett* 2003, **542:**74-78.

22. Yasuda S, Liang MH, Marinova Z, Yahyavi A, Chuang DM: **The mood stabilizers lithium and valproate selectively activate the promoter IV of brain-derived neurotrophic factor in neurons.** *Mol Psychiatry* 2007, **14:**51-59.

23. Kozikowski AP, Chen Y, Gaysin A, Chen B, D'Annibale MA, Suto CM, Langley BC: **Functional differences in epigenetic modulators - superiority of mercaptoacetamide-based histone deacetylase inhibitors relative to hydroxamates in cortical neuron neuroprotection studies.** *J Med Chem* 2007, **50:**3054-3061.

24. Rivieccio MA, Brochier C, Willis DE, Walker BA, D'Annibale MA, McLaughlin K, Siddiq A, Kozikowski AP, Jaffrey SR, Twiss JL et al.: **HDAC6 is a target for protection and regeneration following injury in the nervous system.** *Proc Natl Acad Sci U S A* 2009, **106:**19599-19604.

25. Langley B, D'Annibale MA, Suh K, Ayoub I, Tolhurst A, Bastan B, Yang L, Ko B, Fisher M, Cho S et al.: **Pulse inhibition of histone deacetylases induces complete resistance to oxidative death in cortical neurons without toxicity and reveals a role for cytoplasmic p21waf1/cip1 in cell cycle-independent neuroprotection.** *J Neurosci* 2008, **28:**163-176.

26. Kanai H, Sawa A, Chen RW, Leeds P, Chuang DM: **Valproic acid inhibits histone deacetylase activity and suppresses excitotoxicity-induced GAPDH nuclear accumulation and apoptotic death in neurons.** *Pharmacogen J* 2004, **4:**336-344.

27. Salminen A, Tapiola T, Korhonen P, Suuronen T: **Neuronal apoptosis induced by histone deacetylase inhibitors.** *Mol Brain Res* 1998, **61:**203-206.

28. Suuronen T, Huuskonen J, Pihlaja R, Kyrylenko S, Salminen A: **Regulation of microglial inflammatory response by histone deacetylase inhibitors.** *J Neurochem* 2003, **87:**407-416.

29. Francis YI, Fá M, Ashraf H, Zhang H, Staniszewski A, Latchman DS, Arancio O: **Dysregulation of histone acetylation in the APP/PS1 mouse model of Alzheimer's disease.** *J Alzheimers Dis* 2009, **18:**131-139.

30. Fischer A, Sananbenesi F, Wang X, Dobbin M, Tsai LH: **Recovery of learning and memory is associated with chromatin remodelling.** *Nature* 2007, **447:**178-182.

31. Levenson JM, O'Riordan KJ, Brown KD, Trinh MA, Molfese DL, Sweatt JD: **Regulation of histone acetylation during memory formation in the hippocampus.** *J Biol Chem* 2004, **279:**40545-40559.

32. Lattal K, Barrett RM, Wood MA: **Systemic or intrahippocampal delivery of histone deacetylase inhibitors facilitates fear extinction.** *Behavioral Neuroscience* 2007, **121:**1125-1131.

33. Vecsey CG, Hawk JD, Lattal KM, Stein JM, Fabian SA, Attner MA, Cabrera SM, McDonough CB, Brindle PK, Abel T et al.: **Histone deacetylase inhibitors enhance memory and synaptic plasticity via CREB: CBP-dependent transcriptional activation.** *J Neurosci* 2007, **27:**6128-6140.

34. Hawk JD, Florian C, Abel T: **Post-training intrahippocampal inhibition of class I histone deacetylases enhances long-term object-location memory.** *Learning & Memory* 2011, **18:**367-370.
