# Supplementary material for: HDAC6 as a target for neurodegenerative diseases: what makes it different from the other HDACs?
Source: Mol Neurodegener. 2013 Jan 29;8:7. doi: 10.1186/1750-1326-8-7 (PMC3615964; doi:10.1186/1750-1326-8-7)
Supplement: Additional file 4 — Activity of trichostatin A on HDACs. [file 1750-1326-8-7-S4.docx]

|  |  | **Inhibition of HDAC isoforms** | | | | | | | | | | | | | |
| --- | --- | --- | --- | --- | --- | --- | --- | --- | --- | --- | --- | --- | --- | --- | --- |
| **Sodium butyrate ** |  | **HDACs** | **Class I** | | | | | **Class II** | | | | | | | **Class IV** |
|  |  |  | **HDAC1** | **HDAC2** | | **HDAC3** | **HDAC8** | **HDAC4** | **HDAC5** | | **HDAC7** | **HDAC9** | **HDAC6** | **HDAC10** | **HDAC11** |
|  |  | **IC_50_ (µM)** | 300 [1] | 400 [1] | | - | - | - | - | | 300 [1] | - | - | - | - |
|  |  |  | | | | | | | | | | | | | |
|  |  |  | **Disease** | | **Outcomes** | | | | | **Observed in** | | | | | |
|  |  | ***In vitro* outcomes** | **AD** | | Increased choline acetyltransferase activity [2] | | | | | Cultured rat sympathetic neurons [2] | | | | | |
|  |  |  |  |  | Improvement of aberrant tau phosphorylation [3] | | | | | Human neuroblastoma TR14 cell line [3] | | | | | |
|  |  |  |  |  | Improvement of the APP-CTs induced cytotoxicity [4] | | | | | NGF-Differentiated PC12 cells and rat primary cortical neurons [4] | | | | | |
|  |  |  | **PD** | | Neuroprotection against toxicity of α-synuclein [5] | | | | | Transfected SH-SY5Y cells [5] | | | | | |
|  |  |  |  |  | Neuroprotection against toxicity of MPP^+^ [6] | | | | | Human derived SK-N-SH and rat derived MES 23.5 cells[6] | | | | | |
|  |  |  |  |  | Neuroprotection [7] | | | | | Neuron–glia from F344 rats [7] | | | | | |
|  |  |  |  |  | Neuroprotection against pro-inflammatory stimuli [8] | | | | | Ventral mesencephalic neuron-glia and microglia from F344 rats [8] | | | | | |
|  |  |  | **HD** | | Neuroprotection against oxidative stress [9] | | | | | Cells from rat cerebral cortex [9] | | | | | |
|  |  |  |  |  | Neuroprotection against polyglutamine toxicity [10] | | | | | Transfected MN-1 cells expressing mutant polyglutamine [10] | | | | | |
|  |  |  | **ND**  **and Co** | | Neuroprotection [11] | | | | | Rat cortical neurons [11] | | | | | |
|  |  |  |  |  | Modulation of inflammation [12] | | | | | Murine N9 microglia, rat primary astrocytes, microglia and cerebellar granule cells, rat hippocampal slice cultures [12] | | | | | |
|  |  |  |  |  | Neuroprotection against oxidative stress [13,14] | | | | | Rat cerebral cortex neurons [13],  rat dorsal root ganglion neurons and cortical neurons [14] | | | | | |
|  |  |  |  |  | Neuroprotection [15-17] | | | | | Rat cortical neurons [16], rat astrocytes and cortical neurons [15], rat immature primary cortical neurons [17] | | | | | |
|  |  |  |  |  | Neuroprotection against excitotoxicity [18] | | | | | Rat mature cerebellar granule cells from [18] | | | | | |
|  |  |  |  |  | Induced apoptosis in neuronal cells [19] | | | | | Cerebellar granule neurons from rat and mouse, Neuro-2a neuroblastoma cells [19] | | | | | |
|  |  | ***In vivo* outcomes** | **AD** | | Improvement of learning and memory [20,21] | | | | | Mouse model of AD (APPswe/PS1dE9)[20], APPPS1-21 mice [21] | | | | | |
|  |  |  | **PD** | | Neuroprotection against toxicity of α-synuclein [5] | | | | | Transgenic *Drosophila* [5] | | | | | |
|  |  |  | **HD** | | Extention of survival, improvement of body weight and motor performance [22] | | | | | R6/2 HD mice [22] | | | | | |
|  |  |  |  |  | Neuroprotection against polyglutamine toxicity [23] | | | | | Two *Drosophila* models of polyglutamine disease [23] | | | | | |
|  |  |  |  |  | Improvement of neurological phenotypes [10,24] | | | | | Transgenic mouse model of SBMA [10,24] | | | | | |
|  |  |  | **ND**  **and Co** | | Improvement of learning and memory [25-28] | | | | | CK-p25 Tg mice [25]  Sprague-Dawley rats [26] | | | | | |

Additional file 4. Activity of sodium butyrate on HDACs.

AD: Alzheimer’s disease ; PD: Parkinson’s disease; HD: Hungtington’s disease; ND: neurodegeneration; Co: cognition.

Table references

1. Gurvich N, Tsygankova OM, Meinkoth JL, Klein PS: **Histone deacetylase is a target of valproic acid-mediated cellular differentiation.** *Cancer Res* 2004, **64:**1079-1086.

2. Chireux M, Espinos E, Bloch S, Yoshida M, Weber MJ: **Histone hyperacetylating agents stimulate promoter activity of human choline acetyltransferase gene in transfection experiment.** *Mol Brain Res* 1996, **39:**68-78.

3. Nuydens R, Heers C, Chadarevian A, Dejong M, Nuyens R, Cornelissen F, Geerts H: **Sodium butyrate induces aberrant tau-phosphorylation and programmed cell-death in human neuroblastoma cells.** *Brain Res* 1995, **688:**86-94.

4. Kim HS, Kim EM, Kim NJ, Chang KA, Choi Y, Ahn KW, Lee JH, Kim S, Park CH, Suh YH: **Inhibition of histone deacetylation enhances the neurotoxicity induced by the c-terminal fragments of amyloid precursor protein.** *J Neurosci Res* 2004, **75:**117-124.

5. Kontopoulos E, Parvin JD, Feany MB: **α-Synuclein acts in the nucleus to inhibit histone acetylation and promote neurotoxicity.** *Human Mol Gen* 2006, **15:**3012-3023.

6. Kidd SK, Schneider JS: **Protection of dopaminergic cells from MPP(+)-mediated toxicity by histone deacetylase inhibition.** *Brain Res* 2010, **1354:**172-178.

7. Wu X, Chen PS, Dallas S, Wilson B, Block ML, Wang CC, Kinyamu H, Lu N, Gao X, Leng Y et al.: **Histone deacetylase inhibitors up-regulate astrocyte GDNF and BDNF gene transcription and protect dopaminergic neurons.** *Int J Neuropsychopharmacol* 2008, **11:**1123-1134.

8. Chen PS, Wang CC, Bortner CD, Peng GS, Wu X, Pang H, Lu RB, Gean PW, Chuang DM, Hong JS: **Valproic acid and other histone deacetylase inhibitors induce microglial apoptosis and attenuate lipopolysaccharide-induced dopaminergic neurotoxicity.** *Neuroscience* 2007, **149:**203-212.

9. Ryu H, Lee J, Olofsson BA, Mwidau A, Deodoglu A, Escudero M, Flemington E, Azizkhan-Clifford J, Ferrante RJ, Ratan RR: **Histone deacetylase inhibitors prevent oxidative neuronal death independent of expanded polyglutamine repeats via an Sp1-dependent pathway.** *Proc Natl Acad Sci USA* 2003, **100:**4281-4286.

10. McCampbell A, Taye AA, Whitty L, Penney E, Steffan JS, Fischbeck KH: **Histone deacetylase inhibitors reduce polyglutamine toxicity.** *Proc Natl Acad Sci USA* 2001, **98:**15179-15184.

11. Yasuda S, Liang MH, Marinova Z, Yahyavi A, Chuang DM: **The mood stabilizers lithium and valproate selectively activate the promoter IV of brain-derived neurotrophic factor in neurons.** *Mol Psychiatry* 2007, **14:**51-59.

12. Huuskonen J, Suuronen T, Nuutinen T, Kyrylenko S, Salminen A: **Regulation of microglial inflammatory response by sodium butyrate and short-chain fatty acids.** *Brit J Pharmacol* 2004, **141:**874-880.

13. Langley B, D'Annibale MA, Suh K, Ayoub I, Tolhurst A, Bastan B, Yang L, Ko B, Fisher M, Cho S et al.: **Pulse inhibition of histone deacetylases induces complete resistance to oxidative death in cortical neurons without toxicity and reveals a role for cytoplasmic p21waf1/cip1 in cell cycle-independent neuroprotection.** *J Neurosci* 2008, **28:**163-176.

14. Rivieccio MA, Brochier C, Willis DE, Walker BA, D'Annibale MA, McLaughlin K, Siddiq A, Kozikowski AP, Jaffrey SR, Twiss JL et al.: **HDAC6 is a target for protection and regeneration following injury in the nervous system.** *Proc Natl Acad Sci USA* 2009, **106:**19599-19604.

15. Marinova Z, Leng Y, Leeds P, Chuang DM: **Histone deacetylase inhibition alters histone methylation associated with heat shock protein 70 promoter modifications in astrocytes and neurons.** *Neuropharmacol* 2011, **60:**1109-1115.

16. Marinova Z, Ren M, Wendland JR, Leng Y, Liang MH, Yasuda S, Leeds P, Chuang DM: **Valproic acid induces functional heat-shock protein 70 via class I histone deacetylase inhibition in cortical neurons: a potential role of Sp1 acetylation.** *J Neurochem* 2009, **111:**976-987.

17. Sleiman SF, Berlin J, Basso M, Karuppagounder SS, Rohr J, Ratan RR: **Histone deacetylase inhibitors and mithramycin A impact a similar neuroprotective pathway at a crossroad between cancer and neurodegeneration.** *Pharmaceuticals* 2011, **4:**1183-1195.

18. Kanai H, Sawa A, Chen RW, Leeds P, Chuang DM: **Valproic acid inhibits histone deacetylase activity and suppresses excitotoxicity-induced GAPDH nuclear accumulation and apoptotic death in neurons.** *Pharmacogen J* 2004, **4:**336-344.

19. Salminen A, Tapiola T, Korhonen P, Suuronen T: **Neuronal apoptosis induced by histone deacetylase inhibitors.** *Mol Brain Res* 1998, **61:**203-206.

20. Kilgore M, Miller C, Fass DM, Hennig KM, Haggarty SJ, Sweatt JD, Rumbaugh G: **Inhibitors of class 1 histone deacetylases reverse contextual memory deficits in a mouse model of Alzheimer's disease.** *Neuropsychopharmacol* 2009, **35:**870-880.

21. Govindarajan N, Agis-Balboa RC, Walter J, Sananbenesi F, Fischer A: **Sodium butyrate improves memory function in an Alzheimer's disease mouse model when administered at an advanced stage of disease progression.** *J Alzheimers Dis* 2011, **26:**187-197.

22. Ferrante RJ, Kubilus JK, Lee J, Ryu H, Beesen A, Zucker B, Smith K, Kowall NW, Ratan RR, Luthi-Carter R et al.: **Histone deacetylase inhibition by sodium butyrate chemotherapy ameliorates the neurodegenerative phenotype in Huntington's disease mice.** *J Neurosci* 2003, **23:**9418-9427.

23. Steffan JS, Bodai L, Pallos J, Poelman M, McCampbell A, Apostol BL, Kazantsev A, Schmidt E, Zhu YZ, Greenwald M et al.: **Histone deacetylase inhibitors arrest polyglutamine-dependent neurodegeneration in Drosophila.** *Nature* 2001, **413:**739-743.

24. Minamiyama M, Katsuno M, Adachi H, Waza M, Sang C, Kobayashi Y, Tanaka F, Doyu M, Inukai A, Sobue G: **Sodium butyrate ameliorates phenotypic expression in a transgenic mouse model of spinal and bulbar muscular atrophy.** *Human Mol Gen* 2004, **13:**1183-1192.

25. Fischer A, Sananbenesi F, Wang X, Dobbin M, Tsai LH: **Recovery of learning and memory is associated with chromatin remodelling.** *Nature* 2007, **447:**178-182.

26. Levenson JM, O'Riordan KJ, Brown KD, Trinh MA, Molfese DL, Sweatt JD: **Regulation of histone acetylation during memory formation in the hippocampus.** *J Biol Chem* 2004, **279:**40545-40559.

27. Lattal K, Barrett RM, Wood MA: **Systemic or intrahippocampal delivery of histone deacetylase inhibitors facilitates fear extinction.** *Behav Neurosci* 2007, **121:**1125-1131.

28. Stefanko DP, Barrett RM, Ly AR, Reolon GK, Wood MA: **Modulation of long-term memory for object recognition via HDAC inhibition.** *Proc Natl Acad Sci U S A* 2009, **106:**9447-9452.
