# Supplementary material for: HDAC6 as a target for neurodegenerative diseases: what makes it different from the other HDACs?
Source: Mol Neurodegener. 2013 Jan 29;8:7. doi: 10.1186/1750-1326-8-7 (PMC3615964; doi:10.1186/1750-1326-8-7)
Supplement: Additional file 5 — Activity of sodium butyrate on HDACs. [file 1750-1326-8-7-S5.docx]

|  |  | **Inhibition of HDAC isoforms** | | | | | | | | | | | | |
| --- | --- | --- | --- | --- | --- | --- | --- | --- | --- | --- | --- | --- | --- | --- |
| **Valproic acid**  **** |  | **HDACs** | **Class I** | | | | **Class II** | | | | | | | **Class IV** |
|  |  |  | **HDAC1** | **HDAC2** | **HDAC3** | **HDAC8** | **HDAC4** | **HDAC5** | **HDAC7** | | **HDAC9** | **HDAC6** | **HDAC10** | **HDAC11** |
|  |  | **IC_50_ (µM)** | 1584 [1] | 3068 [1] | 3071 [1] | 7442 [1] | - | - | >10000 [1] | | >10000 [1] | >10000 [1] | - | - |
|  |  |  | 171 [2] | 634 [2] | 5500 [2] | 756 [2] | - | - | - | | - | - | - |  |
|  |  |  | | | | | | | | | | | | |
|  |  |  | **Disease** | **Outcomes** | | | | | | **Observed in** | | | | |
|  |  | ***In vitro* outcomes** | **AD** | Effect on Aβ plaque pathology [3] | | | | | | Human astrocytes [3] | | | | |
|  |  |  | **PD** | Neuroprotection against excitotoxicity [4] | | | | | | Rat cerebellar granule cell and neuron-enriched cerebral cortical cells [4] | | | | |
|  |  |  |  | Neuroprotection against toxicity of MPP^+^ [5] | | | | | | Human derived SK-N-SH and rat derived MES 23.5 cells [5] | | | | |
|  |  |  |  | Neuroprotection [6] | | | | | | Neuron–glia from F344 rats [6] | | | | |
|  |  |  |  | Neuroprotection against pro-inflammatory stimuli [7] | | | | | | Ventral mesencephalic neuron-glia and microglia from F344 rats [7] | | | | |
|  |  |  | **ND and Co** | Neuroprotection [7-11] | | | | | | Rat astrocytes and cortical neurons [8-11]  F344 rat mesencephalic neuron-glia and astroglia [12] | | | | |
|  |  |  |  | Neuroprotection against excitotoxicity [13] | | | | | | Rat mature cerebellar granule cells [13] | | | | |
|  |  | ***In vivo* outcomes** | **AD** | Improvement of learning and memory [14] | | | | | | Mouse model of AD (APPswe/PS1dE9) [14] | | | | |
|  |  |  |  | Effect on Aβ plaque pathology [15] | | | | | | APP23 transgenic mice [15] | | | | |
|  |  |  | **PD** | Protective effect against MPTP-induced PD [16]  Neuroprotection against toxicity of rotenone [17] | | | | | | MPTP mouse model of PD [16]  Rat Model of PD [17] | | | | |
|  |  |  | **ND and Co** | Improvement of long-term memory for extinction of conditioned fear [18] | | | | | | Naive C57BL/6 mice [18] | | | | |
|  |  |  |  | Improvement of 5-lipoxygenase immunoreactive protein in the hippocampus [19] | | | | | | B6129SF2/J mice [19] | | | | |

Additional file 5. Activity of valproic acid on HDACs.

AD: Alzheimer’s disease ; PD : Parkinson’s disease; HD: Hungtington’s disease; ND: neurodegeneration; Co: cognition.

Table references

1. Khan N, Jeffers M, Kumar S, Hackett C, Boldog F, Khramtsov N, Qian X, Mills E, Berghs SC, Carey N et al.: **Determination of the class and isoform selectivity of small-molecule histone deacetylase inhibitors.** *Biochem J* 2008, **409:**581-589.

2. Huber K, Doyon G, Plaks J, Fyne E, Mellors JW, Sluis-Cremer N: **Inhibitors of histone deacetylases.** *J Biol Chem* 2011, **286:**22211-22218.

3. Nuutinen T, Suuronen T, Kauppinen A, Salminen A: **Valproic acid stimulates clusterin expression in human astrocytes: Implications for Alzheimer's disease.** *Neurosci Lett* 2010, **475:**64-68.

4. Leng Y, Chuang DM: **Endogenous α-synuclein is induced by valproic acid through histone deacetylase inhibition and participates in neuroprotection against glutamate-induced excitotoxicity.** *J Neurosci* 2006, **26:**7502-7512.

5. Kidd SK, Schneider JS: **Protection of dopaminergic cells from MPP(+)-mediated toxicity by histone deacetylase inhibition.** *Brain Res* 2010, **1354:**172-178.

6. Wu X, Chen PS, Dallas S, Wilson B, Block ML, Wang CC, Kinyamu H, Lu N, Gao X, Leng Y et al.: **Histone deacetylase inhibitors up-regulate astrocyte GDNF and BDNF gene transcription and protect dopaminergic neurons.** *Int J Neuropsychopharmacol* 2008, **11:**1123-1134.

7. Chen PS, Wang CC, Bortner CD, Peng GS, Wu X, Pang H, Lu RB, Gean PW, Chuang DM, Hong JS: **Valproic acid and other histone deacetylase inhibitors induce microglial apoptosis and attenuate lipopolysaccharide-induced dopaminergic neurotoxicity.** *Neuroscience* 2007, **149:**203-212.

8. Marinova Z, Leng Y, Leeds P, Chuang DM: **Histone deacetylase inhibition alters histone methylation associated with heat shock protein 70 promoter modifications in astrocytes and neurons.** *Neuropharmacol* 2011, **60:**1109-1115.

9. Marinova Z, Ren M, Wendland JR, Leng Y, Liang MH, Yasuda S, Leeds P, Chuang DM: **Valproic acid induces functional heat-shock protein 70 via class I histone deacetylase inhibition in cortical neurons: a potential role of Sp1 acetylation.** *J Neurochem* 2009, **111:**976-987.

10. Jeong MR, Hashimoto R, Senatorov VV, Fujimaki K, Ren M, Lee MS, Chuang DM: **Valproic acid, a mood stabilizer and anticonvulsant, protects rat cerebral cortical neurons from spontaneous cell death: a role of histone deacetylase inhibition.** *FEBS Lett* 2003, **542:**74-78.

11. Yasuda S, Liang MH, Marinova Z, Yahyavi A, Chuang DM: **The mood stabilizers lithium and valproate selectively activate the promoter IV of brain-derived neurotrophic factor in neurons.** *Mol Psychiatry* 2007, **14:**51-59.

12. Chen PS, Peng GS, Li G, Yang S, Wu X, Wang CC, Wilson B, Lu RB, Gean PW, Chuang DM et al.: **Valproate protects dopaminergic neurons in midbrain neuron/glia cultures by stimulating the release of neurotrophic factors from astrocytes.** *Mol Psychiatry* 2006, **11:**1116-1125.

13. Kanai H, Sawa A, Chen RW, Leeds P, Chuang DM: **Valproic acid inhibits histone deacetylase activity and suppresses excitotoxicity-induced GAPDH nuclear accumulation and apoptotic death in neurons.** *Pharmacogen J* 2004, **4:**336-344.

14. Kilgore M, Miller C, Fass DM, Hennig KM, Haggarty SJ, Sweatt JD, Rumbaugh G: **Inhibitors of class 1 histone deacetylases reverse contextual memory deficits in a mouse model of Alzheimer's disease.** *Neuropsychopharmacol* 2009, **35:**870-880.

15. Qing H, He G, Ly PTT, Fox CJ, Staufenbiel M, Cai F, Zhang Z, Wei S, Sun X, Chen CH et al.: **Valproic acid inhibits Aβ production, neuritic plaque formation, and behavioral deficits in Alzheimer's disease mouse models.** *J Exp Med* 2008, **205:**2781-2789.

16. Kidd SK, Schneider JS: **Protective effects of valproic acid on the nigrostriatal dopamine system in a 1-methyl-4-phenyl-1,2,3,6-tetrahydropyridine mouse model of Parkinson's disease.** *Neuroscience* 2011, **194:**189-194.

17. Monti B, Gatta V, Piretti F, Raffaelli S, Virgili M, Contestabile A: **Valproic acid is neuroprotective in the rotenone rat model of Parkinson's disease: involvement of α-synuclein.** Neurotox Res 2010, **17:**130-141.

18. Bredy TW, Barad M: **The histone deacetylase inhibitor valproic acid enhances acquisition, extinction, and reconsolidation of conditioned fear.** *Learn Mem* 2008, **15:**39-45.

19. Yildirim E, Zhang Z, Uz T, Chen CQ, Manev R, Manev H: **Valproate administration to mice increases histone acetylation and 5-lipoxygenase content in the hippocampus.** *Neurosci Lett* 2003, **345:**141-143.
