# Supplementary material for: HDAC6 as a target for neurodegenerative diseases: what makes it different from the other HDACs?
Source: Mol Neurodegener. 2013 Jan 29;8:7. doi: 10.1186/1750-1326-8-7 (PMC3615964; doi:10.1186/1750-1326-8-7)
Supplement: Additional file 6 — Activity of valproic acid on HDACs. [file 1750-1326-8-7-S6.docx]

Additional file 6. HDAC6 specific inhibitors

|  |  |  |  |  |
| --- | --- | --- | --- | --- |
| M344  [1] |  | Thiolate derivative  [2] |  | Mercaptoacetamide derivative [3] |
|  |  |  |  |  |
| Tubacin  [4] |  | Biphenyl containing hydroxamate [5] |  | Phenylisoxazole containing hydroxamate [6] |
|  |  |  |  |  |
| Phenylalanine containing hydroxamate [7] |  | Pyridylalanine containing hydroxamate [7] |  | Tubastatin A  [8] |
|  |  |  |  |  |
| Hydroxamate derivative  [9] |  | Fluorescent hydroxamate derivative [10] |  | WT-161  [11] |

1. Nuutinen T, Suuronen T, Kyrylenko S, Huuskonen J, Salminen A: **Induction of clusterin/apoJ expression by histone deacetylase inhibitors in neural cells.** *Neurochem Int* 2005, **47:**528-538.

2. Suzuki T, Kouketsu A, Itoh Y, Hisakawa S, Maeda S, Yoshida M, Nakagawa H, Miyata N: **Highly potent and selective histone deacetylase 6 inhibitors designed based on a small-molecular substrate.** *Journal of Medicinal Chemistry* 2006, **49:**4809-4812.

3. Kozikowski AP, Chen Y, Gaysin A, Chen B, D'Annibale MA, Suto CM, Langley BC: **Functional differences in epigenetic modulators - superiority of mercaptoacetamide-based histone deacetylase inhibitors relative to hydroxamates in cortical neuron neuroprotection studies.** *J Med Assoc Thai* 2007, **50:**3054-3061.

4. Estiu G, Greenberg E, Harrison CB, Kwiatkowski NP, Mazitschek R, Bradner JE, Wiest O: **Structural origin of selectivity in class II-selective histone deacetylase inhibitors.** *J Med Chem* 2008, **51:**2898-2906.

5. Kozikowski A, Chen Y, Gaysin A, Savoy D, Billadeau D, Kim K: **Chemistry, biology, and QSAR studies of substituted biaryl hydroxamates and mercaptoacetamides as HDAC inhibitors - nanomolar-potency inhibitors of pancreatic cancer cell growth.** *Chem Med Chem* 2008, **3:**487-501.

6. Kozikowski AP, Tapadar S, Luchini DN, Kim KH, Billadeau DD: **Use of the nitrile oxide cycloaddition (NOC) reaction for molecular probe generation: a new class of enzyme selective histone ceacetylase inhibitors (HDACIs) showing picomolar activity at HDAC6.** *J Med Chem* 2008, **51:**4370-4373.

7. Schäfer S, Saunders L, Eliseeva E, Velena A, Jung M, Schwienhorst A, Strasser A, Dickmanns A, Ficner R, Schlimme S et al.: **Phenylalanine-containing hydroxamic acids as selective inhibitors of class IIb histone deacetylases (HDACs).** *Bioorg Med Chem* 2008, **16:**2011-2033.

8. Butler KV, Kalin J, Brochier C, Vistoli G, Langley B, Kozikowski AP: **Rational design and simple chemistry yield a superior, neuroprotective HDAC6 inhibitor, tubastatin A.** *J Am Chem Soc* 2010, **132:**10842-10846.

9. Schlimme S, Hauser AT, Carafa V, Heinke R, Kannan S, Stolfa DA, Cellamare S, Carotti A, Altucci L, Jung M et al.: **Carbamate prodrug voncept for hydroxamate HDAC inhibitors.** *Chem Med Chem* 2011, **6:**1193-1198.

10. Kong Y, Jung M, Wang K, Grindrod S, Velena A, Lee SA, Dakshanamurthy S, Yang Y, Miessau M, Zheng C et al.: **Histone deacetylase cytoplasmic trapping by a novel fluorescent HDAC inhibitor.** *Mol Cancer Ther* 2011, **10:**1591-1599.

11. Guan JS, Haggarty SJ, Giacometti E, Dannenberg JH, Joseph N, Gao J, Nieland TJF, Zhou Y, Wang X, Mazitschek R et al.: **HDAC2 negatively regulates memory formation and synaptic plasticity.** *Nature* 2009, **459:**55-60.
